# Synergising single-cell resolution and 4sU labelling boosts inference of transcriptional bursting

**DOI:** 10.1186/s13059-023-02977-y

**Published:** 2023-06-16

**Authors:** David M. Edwards, Philip Davies, Daniel Hebenstreit

**Affiliations:** School of Life Sciences, University of Warwick, Coventry, UK

**Keywords:** Transcription, Bursting, Dynamics, Inference, Time-resolved, 4sU, Single-cell, Genome-wide, Histone modification

## Abstract

Despite the recent rise of RNA-seq datasets combining single-cell (sc) resolution with 4-thiouridine (4sU) labelling, analytical methods exploiting their power to dissect transcriptional bursting are lacking. Here, we present a mathematical model and Bayesian inference implementation to facilitate genome-wide joint parameter estimation and confidence quantification (R package: burstMCMC). We demonstrate that, unlike conventional scRNA-seq, 4sU scRNA-seq resolves temporal parameters and furthermore boosts inference of dimensionless parameters via a synergy between single-cell resolution and 4sU labelling. We apply our method to published 4sU scRNA-seq data and linked with ChIP-seq data, we uncover previously obscured associations between different parameters and histone modifications.

**Supplementary Information:**

The online version contains supplementary material available at 10.1186/s13059-023-02977-y.

## Background

The canonical understanding of transcription is that it consists of the steps of initiation, elongation and termination. During initiation of transcription in eukaryotes, RNA polymerase (RNAP) is recruited to the promoter via transcription factors (TF), followed by the synthesis of the first few bases of the new transcript [1]. Elongation succeeds initiation, in which RNAP processes along the gene, incorporating RNA nucleotides into the nascent transcript as it progresses [2]. Upon reaching the transcription end site (TES), termination occurs, in which the transcript and RNAP are released from the DNA [3]. Various processing steps take place at different points during transcription to allow for a mature transcript to be produced, including 5′ capping during initiation, splicing to remove intronic (non-coding) sequences during elongation of protein-coding genes, and polyadenylation and cleavage during termination [1–4].

Beyond the general mechanism outlined above, transcription is also a stochastic process subject to intrinsic noise through its fundamental dependence on probabilistic collisions between molecules [5, 6], which are often present in relatively low numbers. Additionally, in many cases, transcription occurs only in short, intense bursts of activity followed by prolonged periods of inactivity, resulting in increased cell-cell variability in transcript counts [7, 8]. Indeed, studies have identified a broad spectrum of genes, from those that are transcribed in a Poissonian fashion, such as housekeeping genes, to those which are very bursty in nature and expressed only in relatively short windows [9, 10]. The transcriptional noise and cell-cell variance induced by bursting can be utilised to, for example, achieve alternative cell fates during differentiation of cell populations without requiring explicit control by genetic programming or external signals [11]. There are several different possible mechanisms thought to contribute to bursting, including the process of reinitiation, in which after transcribing a gene the RNAP is immediately recycled to the transcription start site (TSS) instead of simply terminating and disengaging [12]. This requires looping of the gene to bring the TSS and TES into physical proximity [13], and the link between TSS-TES interactions and bursting has been explored recently [14]. The chromatin state of a gene also plays an important role in governing transcriptional bursting dynamics, which in eukaryotes is dictated largely by histone modifications (HM). Different HMs may result in looser or tighter packing of the chromatin, respectively, with the chromatin density around the TSS being correlated with transcriptional noise [15]. Having active HMs at the TSS results in an increased probability of open chromatin, which facilitates initiation. This is proposed to reduce burstiness, possibly by reducing the duration between active periods [15, 16]. More recent studies have also reported genome-wide direct correlations between the presence of specific HMs at gene promoters and general transcriptional noise [17, 18], while further studies have even linked HMs with the underlying bursting dynamics, both at the individual gene level [19] and genome-wide [20]. Transcriptional bursting in bacteria can also result from supercoiling of the DNA [21]. The proposed mechanism is the accumulation of positive supercoiling caused by the RNAP proceeding through the gene, until it reduces the rate of elongation to the point that it prevents further transcription. Intermittent clearing of supercoiling followed by rapid transcription, and subsequent re-accumulation of supercoiling, results in bursty transcription. Studies have also observed the co-condensation of TFs with transcriptional coactivators such as p300, which mediates cooperative activation of genes by clusters of TFs [22]. This cooperative activation results in non-linear gene regulation and increased burst frequency and burst size for genes enriched in coactivators.

**Fig. 1:**
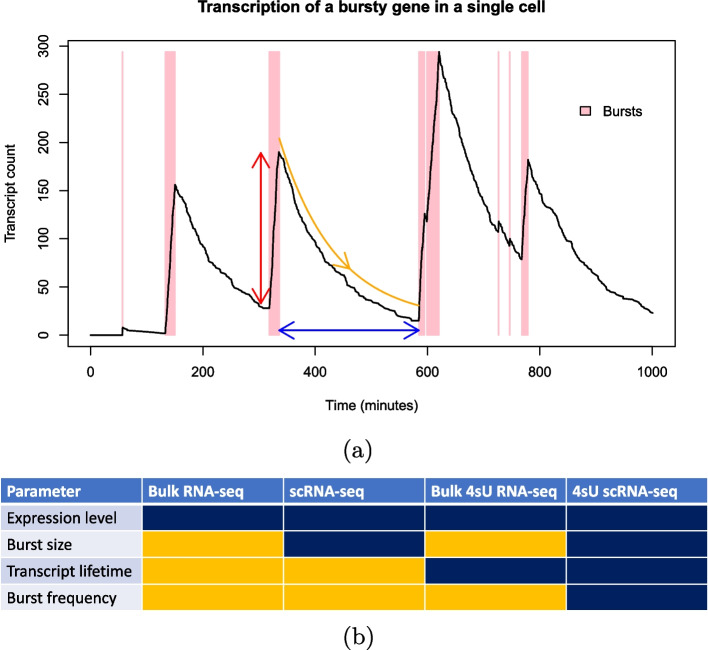
**a** Simulation demonstrating transcriptional bursting for a single gene in a single cell, indicating burst size (red), burst interval (blue, reciprocal of burst frequency), and decay rate (orange, reciprocal of transcript lifetime), while the thickness of the pink shaded regions indicate burst durations. **b** Table showing the parameters governing transcriptional dynamics that can theoretically be obtained using different RNA-seq approaches with no prior information. Dark blue and orange show if a data type does or does not inform a parameter, respectively

Transcriptional bursting may be understood in terms of several parameters (Fig. 1a), including the burst size (transcripts produced per burst, *b*), burst frequency (bursts per unit time, $\kappa$), decay rate (transcripts degraded per unit time, $\delta$), transcript lifetime (average transcript survival time, $\gamma =1/\delta$), burst rate (bursts per transcript lifetime, $a=\kappa /\delta$), and expression level (mean transcripts per cell, $\mu =b\times a$). Many studies make use of fluorescence microscopy-based approaches to interrogate transcriptional bursting dynamics. Single molecule fluorescence in situ hybridisation (smFISH) is a particularly popular approach here although the standard procedure offers only a snapshot of transcript counts across a cell population, with no time-variant information. Therefore, the timescales of bursting events may not be discerned [23], allowing estimation of $\mu$, *b* and *a* but not $\kappa$ or $\delta$. Some smFISH-based experimental set-ups have progressed towards a level of understanding bursting timescales by using hybridisation specific to nascent transcripts [24, 25], although smFISH approaches generally suffer from scalability. While progress is being made towards multiplexing, it can still only analyse a handful of genes at a time compared with sequencing [26–28] or requires complex and labourious set-ups [29]. Sophisticated analysis methods [30] have been developed for time-lapse single-cell RNA imaging data [31] which allows dissection of transcriptional dynamics in great detail, however such approaches are even more limited scale-wise.

Single cell RNA-seq (scRNA-seq) experiments are widely used to analyse genome-wide bursting dynamics. However, scRNA-seq suffers from the same issue as standard smFISH regarding analysis of bursting timescales because it only provides a snapshot of the transcriptomes of a population of cells at a single point in time. Therefore, it has only been possible to obtain burst sizes (*b*) and burst rates (*a*), while burst frequencies ($\kappa$) may not be understood without making assumptions or using prior information on decay rates ($\delta$) measured through separate experiments [10, 32–34]. On the other hand, bulk RNA-seq-based approaches have for several years made use of chemically labelled nucleotides, primarily 4-thiouridine (4sU) as in SLAM-seq, to understand RNA synthesis ($b\times \kappa$) and degradation ($\delta$) rates [35, 36]. The cells are incubated in the presence of 4sU for a given duration, prior to RNA extraction. During this step, 4sU diffuses into the cell nucleus and becomes incorporated into nascently transcribed RNA. Labelled RNA can be bioinformatically distinguished from non-labelled RNA, previously residing in the cell, due to the higher rate of chemically induced cytosine conversion of 4sU relative to regular uracil. Using mathematical modelling, the ratio of labelled to unlabelled transcripts can be used to estimate the turnover rate [37]. However, since bulk RNA-seq neglects the cell-cell variability, it can not be used to study bursting dynamics. Recent advances combine scRNA-seq with 4sU and such datasets have the potential to fully characterise transcriptional bursting dynamics and their timescales (Fig. 1b). Thus far, they have been used for understanding dynamic changes in the transcriptome and/or RNA turnover/splicing rates that occur throughout the cell cycle and cell state transitions [38–42]. Studies with data of this type that have looked at bursting have only done so in a limited manner, using empirically derived statistics as a proxy for burstiness [43], while bursting timescales have remained uncharacterised in recent works [44]. This is despite previous modelling works having shown that degradation is expected to contribute significantly to transcriptional noise and therefore should be accounted for when investigating bursting dynamics [45].

Here, we construct mathematical models to relate observables from 4sU scRNA-seq data to the underlying bursting dynamics and develop an adaptive Markov chain Monte Carlo (MCMC) approach for Bayesian inference of the parameters governing those dynamics. We have produced an R package (https://github.com/hebenstreitLab/burstMCMC) from our method and applied this to published data from [38], demonstrating that we are able to characterise time-resolved transcriptional bursting dynamics for hundreds of genes in parallel. Our approach generates joint probability distributions of the parameters of interest from which estimates can be extracted and confidence in these quantified. This is the first method for joint inference of time-resolved bursting dynamics on a genome-wide scale and is generally applicable to 4sU scRNA-seq datasets. We also show that, even for the dimensionless parameters which can be obtained with conventional scRNA-seq, the accuracy and reliability of estimates can be improved by incorporating the additional information provided by 4sU scRNA-seq. Finally, we build on a previous study which interrogated correlations between bursting parameter estimates and HMs in a genome-wide manner, linking scRNA-seq with ChIP-seq data [20]. Our analysis reveals position-dependent associations between different parameters and HMs only apparent with 4sU scRNA-seq.

## Results

### Model comparison

We tested the advantages provided by 4sU scRNA-seq data coupled with our inference approach over conventional scRNA-seq by comparing our recovery of known bursting parameter values from a simulated dataset using different likelihood functions (Methods). The MCMC algorithm was run five times, using Eqs. 4, 15, 16, 19 and 20 as the likelihood functions, referred to as L1, L2, L1+L2, L3 and L1+L3, respectively.

- L1: The likelihood function of model 1, equivalent to scRNA-seq data without 4sU, relying solely on the UMI counts.
- L2: Equivalent to relying only on single cell T>C conversions, without fully incorporating the UMI counts.
- L1+L2: The likelihood function of model 2, equivalent to 4sU scRNA-seq data, incorporating all of the available information together.
- L3: Equivalent to bulk SLAM-seq data without spike-ins, ignoring UMI counts and using only cell-summed T>C conversions.
- L1+L3: The likelihood function of model 3, equivalent to combining bulk SLAM-seq data without spike-ins and scRNA-seq data.

**Fig. 2:**
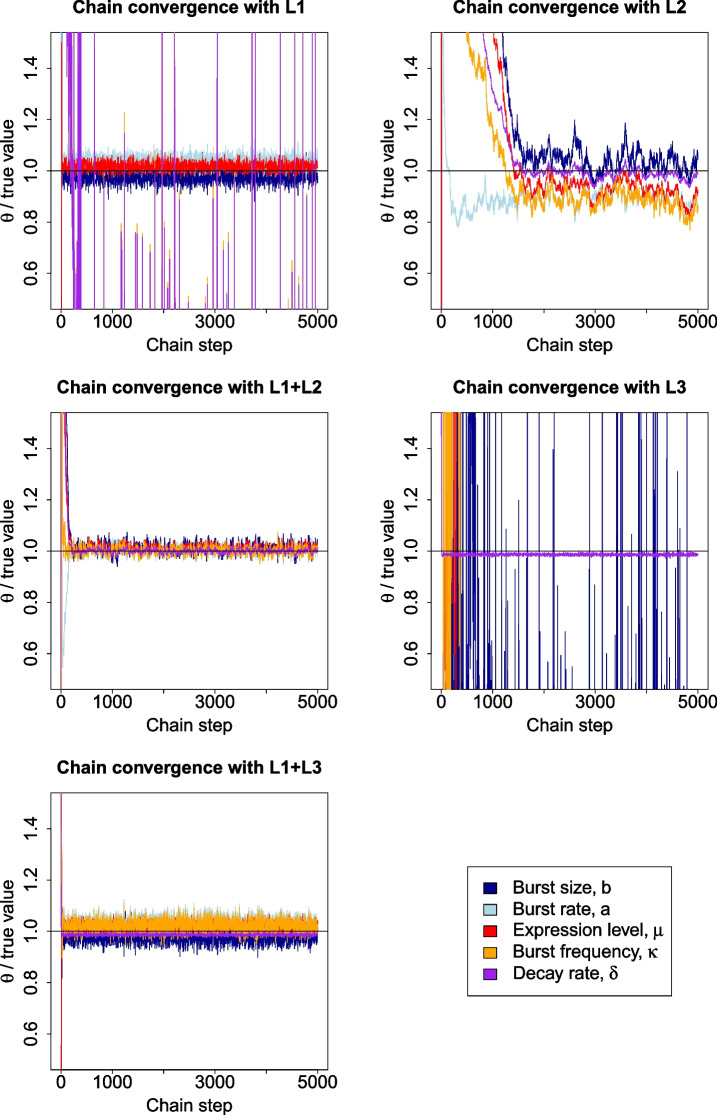
Convergence of Markov chains to true parameter values with simulated data for three different likelihood functions. The parameter values, $\theta$, in the chain are divided by the true value to allow for joint visualisation, with the black horizontal line representing the target value

Convergence to the target distribution is shown in Fig. 2 for each likelihood function, confirming that scRNA-seq data cannot resolve $\kappa$ or $\delta$, but does converge for the other parameters, while L2 and L1+L2 converge for all parameters, confirming that 4sU scRNA-seq data can time-resolve bursting. Unlike L2, L3 is unable to converge for any parameters other than $\delta$, further demonstrating the advantage of cell-specific vs cell-summed T>C conversion data. Conversely, L1+L3 does converge for all parameters, with L1 informing burstiness while L3 informs timescales.

**Fig. 3:**
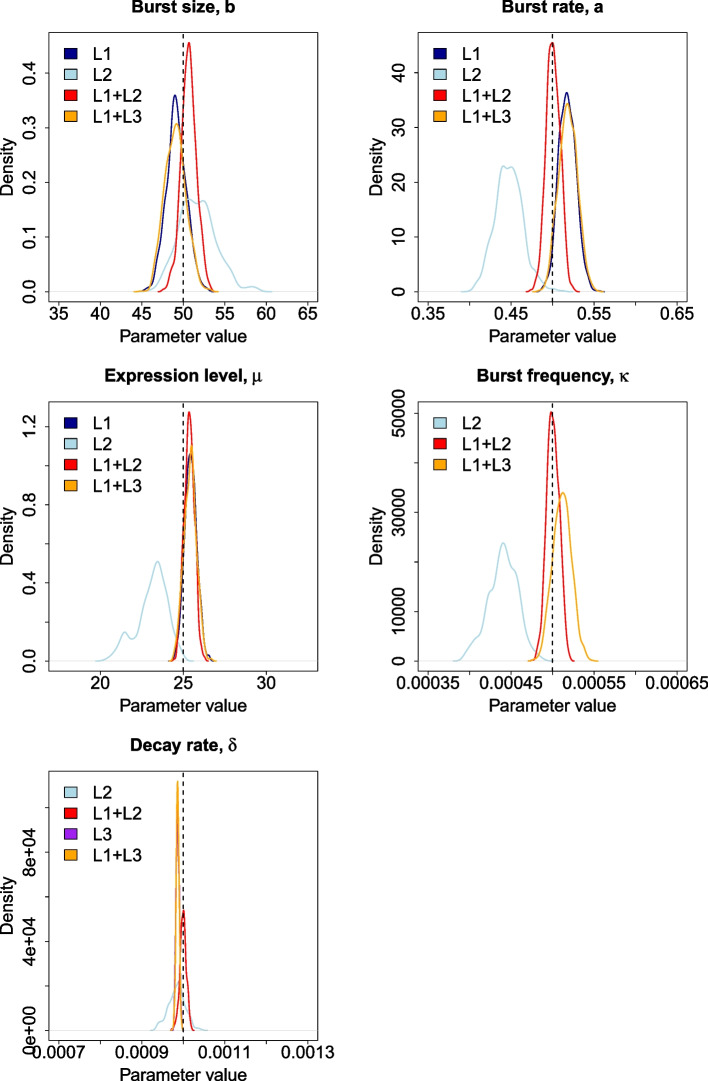
Probability density functions of each parameter derived from posteriors obtained using different likelihood functions, with the dashed black lines representing the true parameter values that were used to simulate the dataset upon which inference was carried out. The densities for $\delta$ obtained with L3 and L1+L3 are difficult to distinguish because they almost perfectly overlap

The resulting posteriors (Fig. 3) indicate that the accuracy and precision of estimates for *a*, *b* and $\mu$ are improved by incorporating the single-cell 4sU conversion data compared to relying solely on scRNA-seq or scRNA-seq with bulk SLAM-seq data, which is because the cell-cell variance in the T>C rate is a function of the transcriptional noise (burstiness) of the gene as well as turnover and, therefore, including such information makes the estimation more robust. Likewise, we see that while conventional scRNA-seq may not resolve $\kappa$ or $\delta$, including the UMI count information with the conversion data also results in more precise and accurate estimates of these parameters. This is because the set of T>C conversions is a function of *a*, *b* and $\delta$, while the UMI counts are a function of *a* and *b*. Therefore, including the UMI data improves inference of *a* and *b*, which reduces the error associated with $\delta$ in our joint inference approach.

Overall, we see that L1+L2 outperforms all other likelihood functions for all parameters including L1+L3, demonstrating the benefits that a fully integrated analysis of time-resolved bursting dynamics using 4sU scRNA-seq data provides over more limited, separate treatments of subsets of the parameters by combining scRNA-seq (*a* and *b*) and bulk SLAM-seq ($\delta$) information. This is apparent in this example of a gene with moderate expression, high transcriptional noise and a transcript lifetime similar to the 4sU pulse duration.

### Inference on data from Qiu

We next applied our method to 4sU scRNA-seq data published in 2020 by Qiu et al, which used human K562 cells [38]. Inference on the data from Qiu was carried out for all genes with at least one read and observed T>C conversion in both the 4sU and control datasets, running the MCMC algorithm in parallel on each to obtain a posterior from model 2 or model 3 if required (Methods). The final set of genes to be analysed was selected based on those with sufficient confidence in all parameter estimates. Therefore, a maximum CV value of 0.45 was imposed for all parameter estimates, so that only genes with no CV $>0.45$ would be included, leaving 584 genes as the final selected set.

**Fig. 4:**
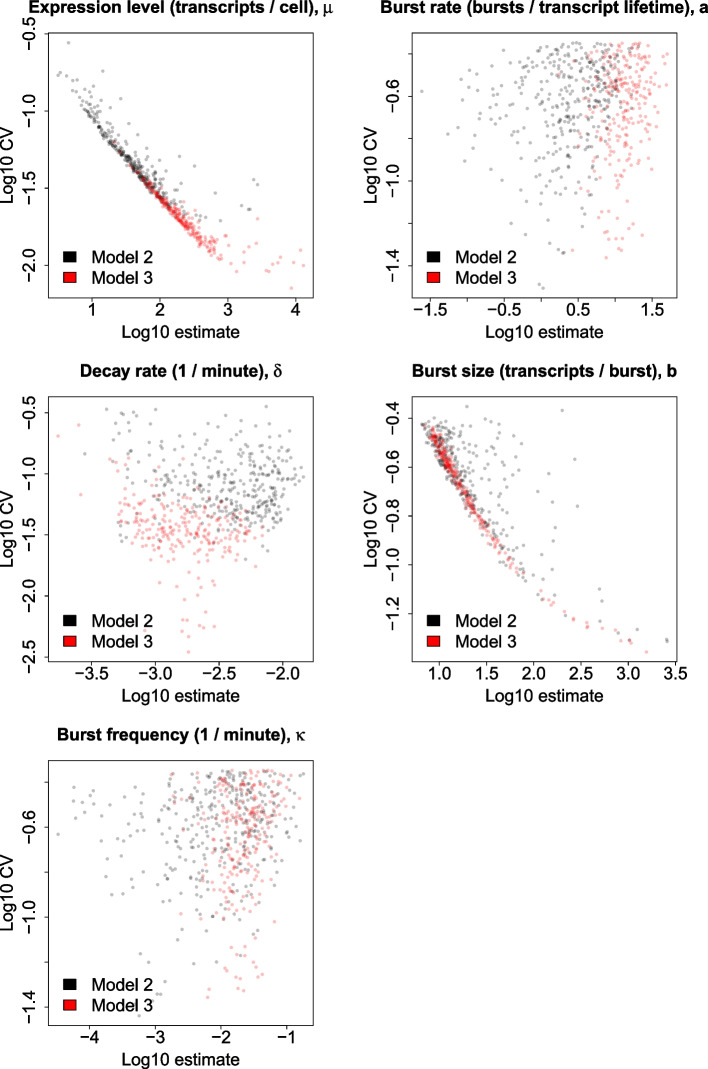
Estimates vs CVs of all parameters derived from sampled posteriors for all 584 selected genes, with those obtained using models 2 or 3 displayed in black or red, respectively

For the selected genes we observe that the quality of our estimates depends upon the location of the gene within parameter space, as shown in Fig. 4, which depicts estimate vs CV for all parameters. $CV(\delta )$ has an optimal (minimum) value for $\delta$ corresponding to an average transcript lifetime equal to the 4sU pulse duration (4 hours), with confidence decreasing bidirectionally and outliers with very low $CV(\delta )$ corresponding to genes with $\mu \ge 1000$. We also have increased confidence in general for genes with higher $\mu$ since estimates for such genes are informed by a greater volume of data. Likewise, genes with greater *b* have greater confidence because, firstly, increased *b* results in higher $\mu$. Secondly, for a given $\mu$, having a higher *b* implies lower *a*, meaning that the transcriptional noise is higher, resulting in a more heavily skewed transcript count distribution (across cells) which may be more precisely attributed to a region of parameter space. We do not see a visually obvious trend in confidence for *a*. This is because it is associated with higher expression level but lower transcriptional noise. Therefore, a gene with higher *a* has more data points with which to inform the estimate but a less skewed transcript count distribution, so that the effects on confidence tend to cancel each other out. The trend in confidence for $\kappa$ is essentially dictated by the *a* and $\delta$ values for the gene.

Instead of relying solely on model 2, for some genes we must switch to an alternative (model 3). This occurs when genes lie within a region of parameter space such that the solution to Eq. 9 becomes unstable. Figure 4 provides evidence supporting the reliability of our inference approach, since the model 2 and 3 genes generally occupy the same regions of the plot and exhibit the same relationships between confidence and estimate for each parameter. This also illustrates the increased probability for a gene to reside within unstable parameter space, and therefore require use of model 3, when $\mu$ and *a* are higher and when $\delta$ is lower.

We reinforce our results by demonstrating a strong positive correlation about the diagonal between our estimates of $\delta$ and cell-matched values calculated in [36] for the same genes (Additional file 1: Fig. S3). Further assessment of our parameter estimation and confidence quantification was provided by carrying out inference on simulated data. This simulation-based validation differs from the previously described model comparison analysis (Figs. 2 and 3) in that experimental settings, such as cell number, cell capture efficiency and sequencing depth, were equivalent to those in the Qiu dataset rather than being idealised, and the bursting parameter values estimated for each of the 12276 genes we analysed were used as the true values for a corresponding simulated gene. Strong, tight correlations about the diagonal between estimates and true parameter values confirmed the capacity for the algorithm to recover known parameter values (Additional file 1: Fig. S4).

**Fig. 5:**
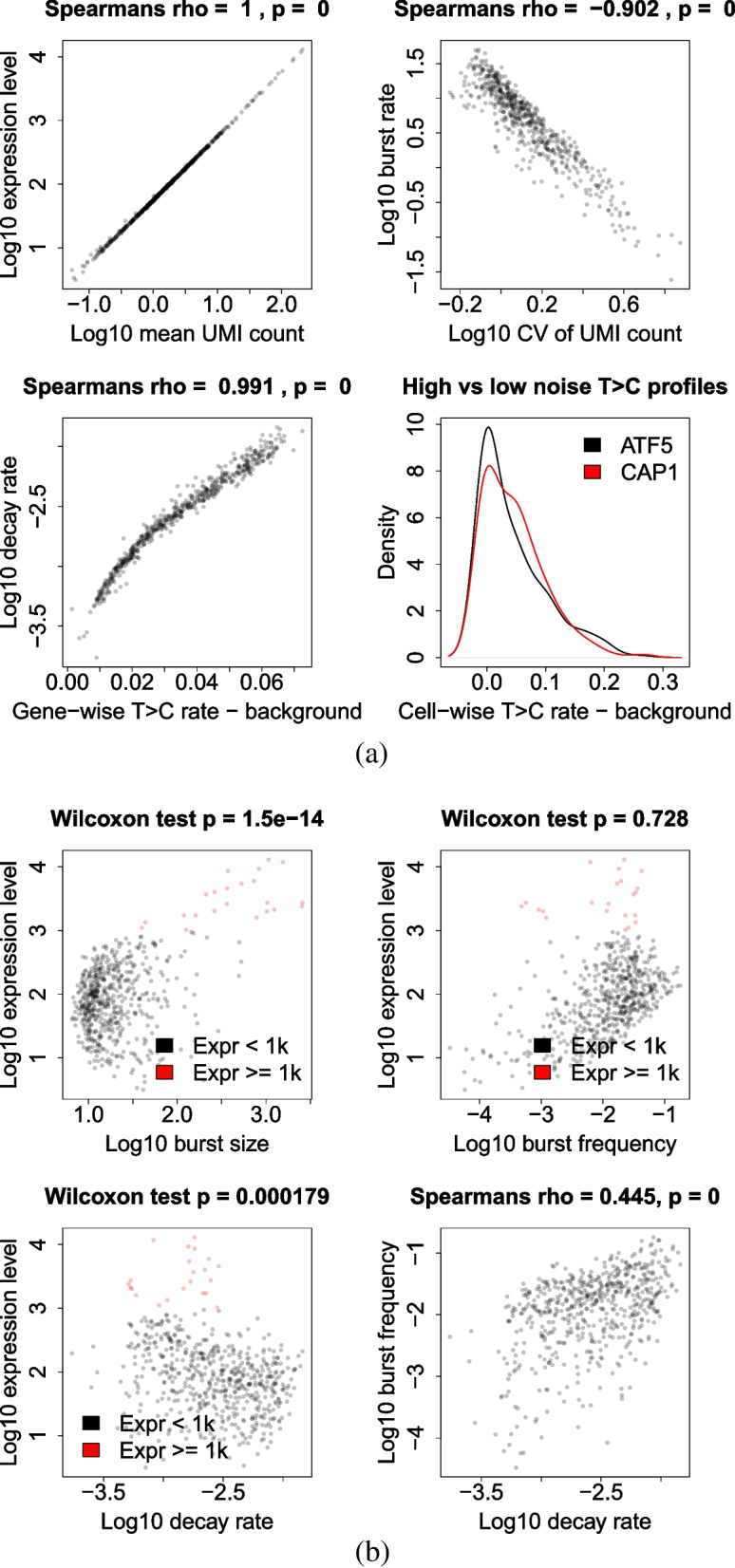
**a** Correlations between statistics of the observable data and related bursting parameter estimates, with Spearman’s rank correlation strength (rho) and statistical significance (p) displayed. Bottom right compares the cell-specific T>C rates minus gene-specific background for the *ATF5* and *CAP1* genes, which are expressed with high and low noise, respectively. **b** Estimates for different parameters plotted against each other. Statistical significance of difference in *b*, $\kappa$ and $\delta$ for genes with very high ($\mu \ge 1000$) expression level vs other genes ($\mu <1000$) is shown with the p-value calculated using the Wilcoxon test. Also shown in the bottom right is the Spearman’s rank correlation strength (rho) and statistical significance (p) of $\kappa$ against $\delta$

Now that we have estimates for all parameters of interest, it is possible to demonstrate how the different aspects of the data feed into informing the joint probability distribution. Figure 5a illustrates some expected correlations, showing that $\mu$ correlates very strongly with the mean UMI count and that $\delta$ correlates very strongly with the 4sU - control T>C rate, since these values reflect the overall activity and turnover of the gene, respectively. We see that *a* correlates strongly against the CV of the UMI count, which reflects the relationship between bursting and cell-cell variability. It is also possible to demonstrate the aforementioned complex relationship between burstiness and the shape of the single-cell T>C count data, but not in a genome-wide manner since the effect is masked by variation in $\mu$ and $\delta$. Therefore, we instead compare a pair of genes (*ATF5* and *CAP1*) with very similar estimates for $\mu$ and $\delta$ but very different values of *a* (and therefore also *b* and $\kappa$), with *ATF5* being expressed in a far more bursty fashion. The estimates for the different parameters of these genes are given by Table 1.

**Table 1:** Parameter estimates for the *ATF5* and *CAP1* genes

	ATF5	CAP1
*b*	66.1	10.2
$\mu$	62.5	66.1
$\kappa$	0.00426	0.0289
$\delta$	0.00447	0.00409
*a*	0.957	7.07

The density plot in Fig. 5a compares the distribution of cell-specific T>C rates (minus gene-specific background) across all reads in the cell for the aforementioned pair of genes. There is a clear difference in the shape of the distribution, with the bursty gene having a greater density at either extreme while the gene with less noisy expression has a greater intermediate density. This is because large, infrequent bursting has a binarising effect, meaning that most cells either have a low or high T>C rate. Those with a low rate correspond to those which have had no bursts occur during the 4sU pulse, resulting in their entire transcript population comprising those surviving from before the pulse. Those with a high rate correspond to those which have had at least one burst occur during the pulse. Since the bursts tend to be large, this results in the majority of the transcript pool being comprised of newly synthesised transcripts. On the other hand, smaller, more frequent bursts causes the surviving transcripts to gradually become replaced by new transcripts in a more uniform manner across cells. Similarly to how scRNA-seq reveals differences in cell-cell variation in transcript counts for two genes with otherwise equal expression levels, 4sU scRNA-seq also reveals differences in cell-cell variation in new transcript proportions for two genes with otherwise equal decay rates.

Despite controlling for $\mu$ and $\delta$ in this pairwise comparison of a high vs low noise gene, the effect of bursting on cell-specific T>C rates shown in Fig. 5a is still somewhat obscured by the variable cell-specific capture efficiencies, $\alpha$, present in the data. Therefore, datasets were simulated in the same manner as for the model comparison analysis, except $\lambda _s=0.001$, and $\alpha =1$ to totally control for the effect of capture efficiencies. Datasets were simulated for a gene with high noise and another with low noise with parameter values set as shown in Table 2.

**Table 2:** Parameter values for simulated high and low noise genes

	High noise	Low noise
*b*	250	25
$\mu$	250	250
$\kappa$	0.001	0.01
$\delta$	0.001	0.001
*a*	1	10

The differential transition from surviving to new transcript pool for high and low noise genes is demonstrated in Additional file 2, which shows the cell-specific T>C rate distributions for data simulated with different pulse durations. This illustrates the previously discussed effect of bursting on cell-cell turnover variation more clearly, visualising the bimodal vs unimodal transitions occurring under high vs low noise conditions with a video.

#### Biological findings

Correlating the parameter estimates against each other for our 584 genes also reveals that genes with extremely high expression levels, the majority of which are mitochondrial genes, are able to achieve these high levels primarily by having very large bursts, rather than very frequent bursts or very stable transcripts, although the decay rates do appear somewhat constrained (Fig. 5b). There may be biological upper limits on $\kappa$ due to the various factors required to be in place to prime a gene for activity and, therefore, it may be preferable to instead increase burst duration (reduce $k_{off}$), and therefore burst size, for very high expression levels [32]. A similar phenomenon has been observed previously, in which *MYC* overexpression lead to increased expression in target genes through increased burst duration and size, rather than increased burst frequency [46, 47]. Estimates for $\kappa$ and $\delta$ are also positively correlated, despite $\kappa$ and $\delta$ varying across several orders of magnitude. This correlation may be the result of a selective pressure to limit the variability of *a* and, for example, prevent transcriptional noise levels from becoming excessively high. Alternatively, high burst frequencies would correspond to RNAP rapidly processing over the gene, allowing less time to pause for the nascent transcript to be folded/spliced appropriately than with lower burst frequencies [2], resulting in reduced transcript stability.

### Histone modifications and bursting

**Fig. 6:**
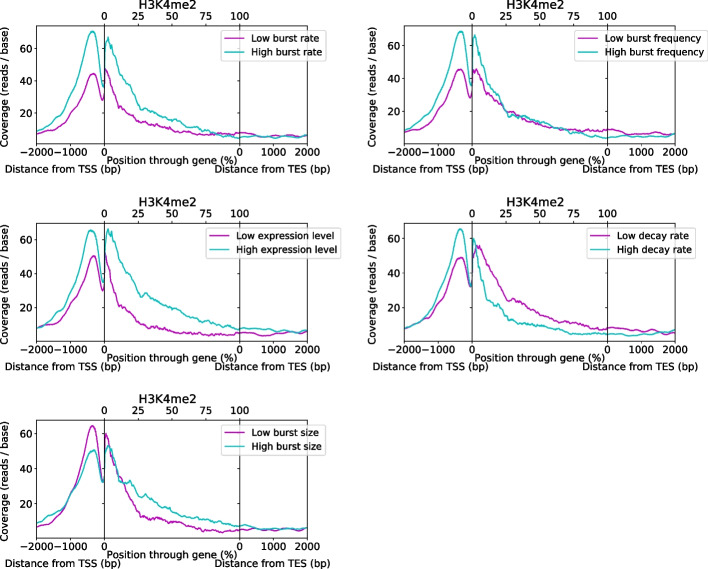
Metagene plots of H3k4me2 coverage, comparing profiles for the top and bottom 50% of genes when split according to their estimates for each parameter, denoted by high and low, as indicated

We next explored the relationship between HMs and transcriptional bursting dynamics with a metagene analysis carried out using ChIP-seq data for eight previously analysed HMs [20] and two further HMs (Methods). In this analysis, we removed mitochondrial genes and genes for which we lacked HM data from our set with high confidence parameter estimates, with 505 genes ultimately being included. Of the eight previously studied HMs we analysed, the profiles generally fall into the two previously described categeories [20], being either predominantly promoter-localised (H3K4me2, H3K4me3, H3K9ac, H3K27ac, Fig. 6 and Additional file 1: Figs. S6-8) or gene body (GB)-localised (H3K4me1, H3K36me3, H3K79me2, H4K20me1, Additional file 1: Figs. S9-13). To better understand the association between HM profile and bursting parameters, the genes were split in half, sorted by parameter estimate for each of the five parameters. Metagene comparison reveals position-dependent associations for promoter-localised HMs, using H3K4me2 as an example (Fig. 6). It appears that HM presence at the promoter and through the GB is associated with increased $\mu$ and also *a*, while increased $\kappa$ is specifically associated with promoter but not GB presence. Conversely, presence through the GB excluding the promoter region appears associated with increased *b* and reduced $\delta$.

This analysis builds upon a previous scRNA-seq study which correlated bursting parameter estimates with HM localisation by averaging the ChIP-seq coverage from 2000 bp upstream of the TSS to the TES for each gene [20]. They were unable to obtain estimates of $\kappa$ or $\delta$ due to a lack of published data on transcript turnover rates for the cell type (hESCs). Our results are in agreement with [20] despite having a different cell type, but additional complexities are revealed which are only apparent with our metagene analysis combined with the capacity to estimate $\kappa$ and $\delta$ afforded by 4sU scRNA-seq. For promoter-localised HMs, they report positive associations between HM presence and both *a* and *b*, whilst we demonstrate that the association with *b* is specific to the GB. We confirm that the association with *a* holds throughout both the promoter and GB, but show that this is a result of a promoter-specific positive $\kappa$ association and a GB-specific negative $\delta$ association, thereby further demonstrating the advantages of 4sU scRNA-seq inference.

**Fig. 7:**
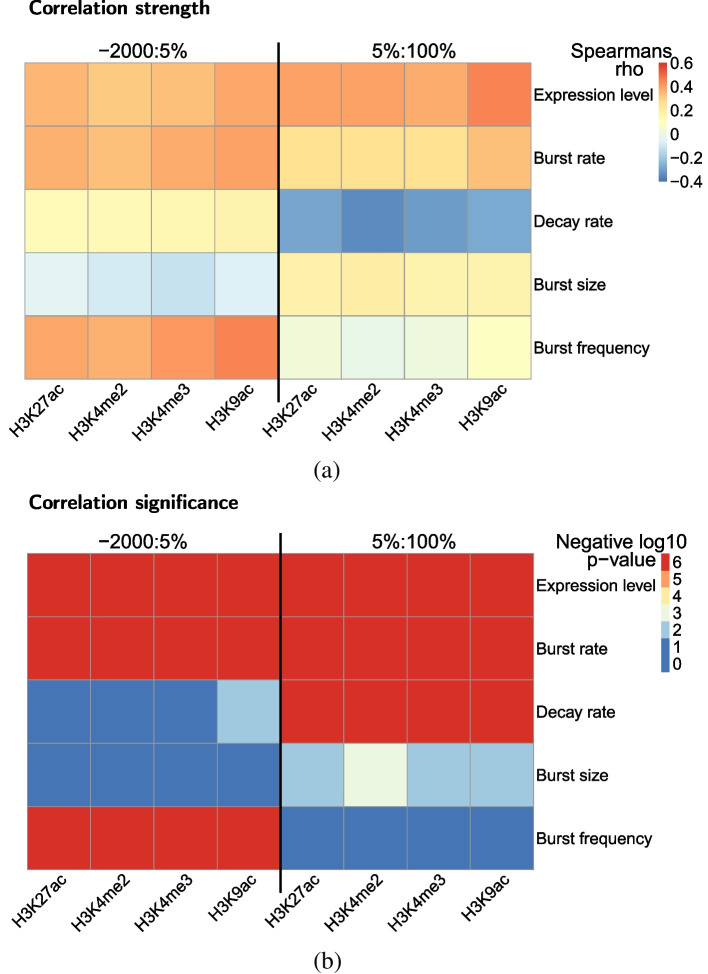
**a** Heatmap showing the Spearman’s rank rho as the heat intensity value for the correlations between bursting parameter estimates and the mean promoter-localised HM coverage values across the -2000:5% and 5%:100% regions. More intense red or blue colouration indicates a stronger positive or negative correlation, respectively, while neutral indicates no/weak correlation. **b** Heatmap showing the Spearman’s rank *p*-value (adjusted for multiple hypothesis testing) as the heat intensity value for the correlations between bursting parameter estimates and the mean promoter-localised HM coverage values across the -2000:5% and 5%:100% regions. The heat values are discretised, corresponding to negative log10 *p*-value thresholds. For example, the most intense blue indicates that, for the given correlation, $10^{-2}<p$, meaning no statistical significance, the neutral colour indicates that $10^{-4}<p\le 10^{-3}$, while the most intense red indicates that $p\le 10^{-6}$

In order to statistically test these apparent associations, the average HM coverage values around the promoter and through the GB excluding the promoter were obtained for each HM (Methods), taking the average value from 2000 bp upstream of the TSS to 5% through the GB (-2000:5%) and from 5% through the GB to the TES (5%:100%), respectively. Spearman’s rank correlation of the mean value for each promoter-localised HM against each parameter across our 505 genes confirmed the direction and quantified the strength (Fig. 7a), as well as confirmed the statistical significance of the suspected associations (Fig. 7b).

The association between promoter-localised HM presence and reduced decay rate is consistent with previous reports of a link between HMs and pre-RNA processing. The RNAP elongation speed may be modulated by HMs or they may be responsible for the recruitment of splicing factors [48, 49]. This could result in more stable RNA by ensuring correct splicing and/or polyadenylation. GB presence of promoter-localised HMs could also result in increased burst size by facilitating TSS-TES contact through the maintenance of the open chromatin state around the TES. Coupled with the free movement of RNAP through the GB, this may increase the burst size by allowing RNAPs to quickly and repeatedly generate multiple transcripts by promoting polymerase recycling [14]. Another hypothesis is the presence alternative TSSs in the GB, which may transcribe simultaneously upon gene activation, causing increased burst size, with 483 out of the 505 analysed genes exhibiting alternative TSSs according to the gtf. Metagenes for the rest of the aforementioned HMs along with the correlation/statistical analysis of the GB-localised HMs can be found in Additional file 1, as well as analysis of two additional HMs (H3K18ac and H4K16ac) not analysed in [20] (Additional file 1: Figs. S14-16), but which were shown to be strongly linked to active enhancer regions [50].

## Discussion

With the inference approach presented here, we demonstrate the capacity to obtain genome-wide estimates of the parameters governing transcriptional bursting dynamics and the timescales upon which they occur from a single dataset with no prior knowledge. By sampling from the full joint probability distributions of the parameter values given the data, we are able to quantify confidence in our estimates and take into account the complex interdependencies between the different parameters and 4sU scRNA-seq data, revealing the regions of parameter space for which we have the most accurate and precise estimates. We show that the distribution of 4sU-induced T>C conversions across cells is shaped not only by the turnover rate and expression level of the gene but also by the transcriptional noise and that this information can therefore be used to improve estimates of burst rate (*a*) and burst size (*b*) beyond the level obtainable with conventional scRNA-seq. In this way, combining metabolic labelling and single cell resolution has an effect greater than the sum of their parts on inference power. Previous analysis of transcriptional bursting using 4sU scRNA-seq data has tapped into this idea by estimating the proportion of new transcripts (based on T>C conversions) in each cell for a particular gene and then using the standard deviation of this new to total ratio as a proxy for burstiness [43]. However, as clearly demonstrated by the video in Additional file 2, this distribution, and therefore its standard deviation, is shaped not only by transcriptional noise but also by RNA turnover and may be skewed by technical noise such as variation in capture efficiency. Therefore, along with the overall expression level, this needs to be explicitly accounted for in order to accurately quantify burstiness, as is naturally achieved with our mathematical model.

Having genome-wide estimates of the parameters governing transcriptional dynamics means that it is possible to use the variation which naturally exists between genes to examine the relationships between the different parameters and other features, such as HMs, instead of having to rely on experiments which artificially perturb the cells to gain insight via a single gene system. In agreement with previous reports [42], we find that the genes with very high expression levels are primarily mitochondrial genes. Going beyond this, we show that such activity levels are achieved by having large burst sizes rather than increased RNA stability or burst frequency, which we hypothesise could be due to biological constraints on the rate of switching between active and inactive states [32], potentially making it favourable to instead increase the duration of bursts, and therefore the burst size, as has similarly been observed for *MYC*-driven transcription [46, 47]. Whereas some studies have found the variation in decay rates (in mESCs) across genes to be an order of magnitude lower than for the other parameters, and therefore negligible [32], we found significant variation in K562 cells which was important to account for in order to properly estimate burst frequencies. This is in line with previous predictions that transcript stability plays an important role in modulating gene expression noise [45]. Indeed, our analysis revealed an unexpected positive correlation between burst frequency and decay rate, resulting in the burst rate, and therefore transcriptional noise, being constrained. One may speculate that only noise levels within a certain range are tolerated, with extreme values resulting in too few cells expressing the gene for a given function to be achieved, such as the appropriate proportion of cells in an isogenic population undergoing differentiation [11, 51], manifesting as the observed correlation. A mechanistic, rather than evolutionary, explanation is that high burst frequencies result in rapid flux of RNAP through the gene, such that less time is allowed for pausing, during which appropriate folding and/or splicing of the nascent transcript is facilitated [2]. This would would reduce transcript stability and cause the observed correlation.

Examining the relationship between bursting parameters and HMs genome-wide produced results consistent with but advancing upon previous work [20]. Combining our metagene analysis with the additional information provided by 4sU scRNA-seq over inference on conventional scRNA-seq reveals intricacies that were not previously apparent. The presence of GB-localised HMs throughout the gene is generally associated with increased burst rate (bursts per transcript lifetime) via increased burst frequency (bursts per minute), while promoter-localised HMs are only associated with increased burst frequency when found around the TSS. Their presence further downstream remains associated with increased burst rate, and therefore reduced transcriptional noise, but through reduced decay rate rather than increased burst frequency. The association with reduced decay rate may be related to the previously documented influence of HMs on pre-RNA processing, which is achieved, for example, by modulating RNAP elongation speed and/or by recruiting splicing factors [48, 49]. This may increase RNA stability by reducing the probability of incorrect splicing or polyadenylation. Presence of promoter-localised HMs throughout the GB but not at the TSS is also associated with increased burst size. Downstream presence could facilitate interactions between the TSS and the TES by maintaining the open chromatin state around the TES. This, along with maintaining the free movement of RNAP through the GB, could promote polymerase recycling and therefore increased burst size by allowing RNAPs to quickly and repeatedly fire off multiple transcripts during an active period [14]. Another possible explanation for the association between promoter-localised HM presence in the GB with burst size is that there are multiple, alternative TSSs found within genes. These may initiate transcription in a non-independent manner, leading to increased burst size when there are more/stronger alternative TSSs, as signalled by HM presence.

The inference approach described here is generally applicable to 4sU scRNA-seq datasets which have RNA spike-ins and UMIs for any organism or cell type. Furthermore, the model could easily be expanded to integrate an arbitrarily large number of repeat experiments by extending the Markov chain according to the product of the likelihood functions of each dataset. Indeed, such a scheme which utilised datasets with different 4sU pulse durations could theoretically characterise the transcriptional dynamics of all genes genome-wide. For example, inference carried out using two datasets with long and short pulse durations would facilitate estimates for genes with long and short transcript lifetimes, respectively, along with everything in between. A caveat of our analysis is the asynchronisation of the cell cycle phase across the population. This may confound the results in two ways, firstly because different phases have a different cellular environment, influencing the global transcriptional dynamics and causing variation in the underlying parameter values for the same gene between cells in different phases. Secondly, there is variation in the copy number of genes throughout the cell cycle, with an unknown proportion of cells having one or two copies of each nuclear gene. Confounding effects on the inference could be resolved by separation of the different subpopulations of cells by cell cycle phase using, for example, fluorescence-activated cell sorting prior to sequencing [33], and/or by using allele-specific/sensitive scRNA-seq approaches combined with metabolic labelling [17, 34]. As 4sU scRNA-seq data becomes more common place and there are improvements in capture efficiencies, sequencing depths and cell numbers, it will be possible to robustly infer time-resolved transcriptional bursting dynamics for a far greater number of genes from a single experimental set up. Our findings on burst dynamics and their associations with HMs could be a valuable starting point to inform future experimental work investigating this area, while further application of our method beyond what is presented here might hint at other, novel mechanistic relations.

## Conclusions

In conclusion, we have developed a mathematical model to maximally exploit the power of 4sU scRNA-seq datasets to examine transcriptional bursting, tapping into the synergy between single-cell resolution and 4sU labelling which manifests in the cell-specific T>C rate distributions. The advantages over conventional scRNA-seq were demonstrated in detail using small-scale simulations and performance of the algorithm across parameter space was validated with large-scale simulations. We applied our inference approach to published 4sU scRNA-seq data to obtain genome-wide joint parameter estimates and confidence quantifications, finding an unexpected correlation between burst frequency and decay rate, and that genes with extremely high expression levels achieve this primarily through increased burst size. Finally, we linked our estimates with published ChIP-seq data, revealing position-dependent associations between different histone modifications and parameter estimates which only become apparent with 4sU scRNA-seq as opposed to conventional scRNA-seq.

## Methods

### Data processing and analysis

#### 4sU scRNA-seq

**Fig. 8:**
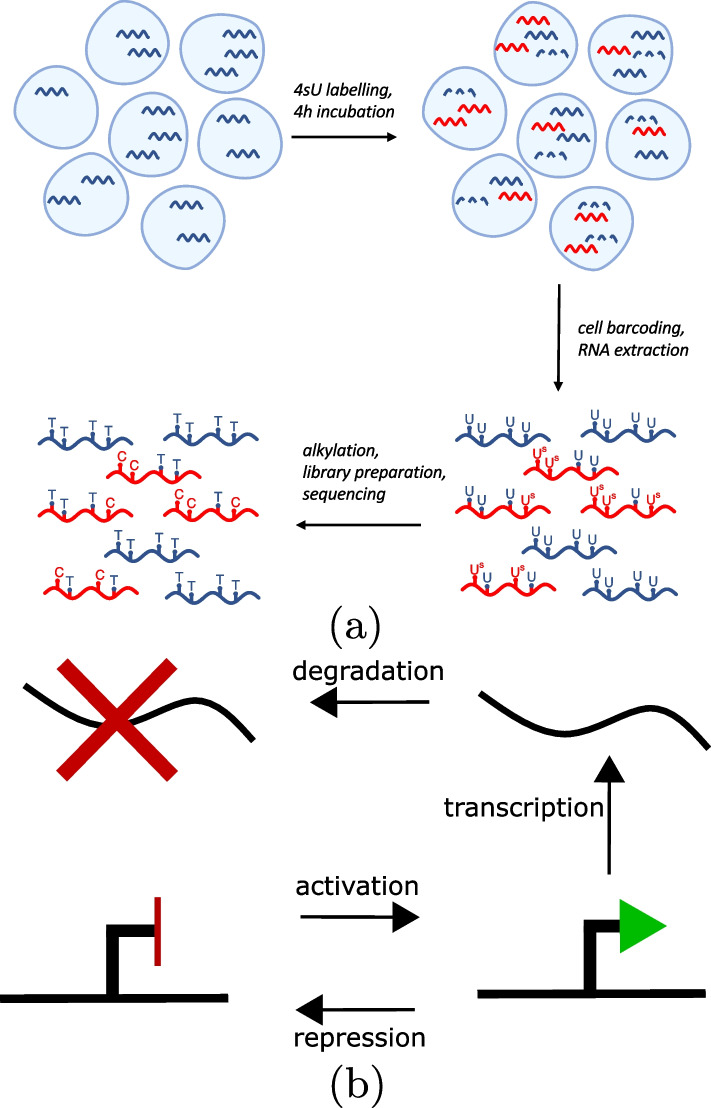
**a** Cells with variable mRNA content (blue lines) shown for an example gene. Cells are incubated in the presence of the uracil analogue 4sU for a set amount of time (4h). Transcripts that are produced during that period (red) become labelled with 4sU, which is incorporated instead of uracil. During the incubation period, natural mRNA decay also takes place (dashed lines). Following cell barcoding and RNA extraction, the RNA is chemically treated, resulting in the modification (alkylation) of the 4sU moieties incorporated into all labelled transcripts (U^S^). In turn, this introduces T-to-C base flip mutations at the points of 4sU incorporation during the first stage of cDNA library preparation (reverse transcription), which are subsequently detected by sequencing. **b** Schematic representation of the two state model, with the four reactions (activation, repression, transcription and degradation) acting on the three species (repressed gene, active gene and transcript)

The main datasets that were used for parameter inference in this study were produced in Qiu et al 2020 [38], downloaded from the GEO series GSE141851. Two datasets from this series were used, both using K562 cells; a negative control dataset with TFEA chemical conversion treatment but with no 4sU added, and another dataset which had 4sU added 4 h before chemical treatment, with GEO sample IDs GSM4512696 and GSM4512697, respectively. These are Drop-seq datasets and thus were processed according to the “Drop-seq alignment cookbook” (https://mccarrolllab.org/wp-content/uploads/2016/03/Drop-seqAlignmentCookbookv1.2Jan2016.pdf). A custom Python script was used to carry out trimming of read pairs with any base with phred quality $\le 10$, and to clip adaptor and polyA tail sequences. The trimmed reads were then aligned to the primary human genome assembly (GRCh38.p13), the fasta file for which was obtained from gencode (https://www.gencodegenes.org/human/), using bwa to build the genome index and for the actual alignment [52]. Custom Python scripts were then used to map the aligned reads with mapq score $\ge 10$ to their genes according to the gencode.v36 primary human genome assembly annotation gtf file, before extracting cell-specific (using the cell ID part of the read 1 barcode) UMI counts and total read counts for each gene, along with gene-specific, cell-specific information for each read about the number of genomic T bases (found in the fasta sequence across the aligned read positions) and the number of those which were converted to C bases in the read sequence. Cell selection was then carried out to exclude those cell IDs corresponding to empty droplets by ordering the cell IDs based on the total number of corresponding read pairs and then selecting the top 400 or 795 IDs for the control and 4sU dataset, respectively, as specified in [38]. The control dataset was then used to derive the gene-specific background T>C conversion rates, $\lambda _s$, based on the proportion of genomic Ts which were converted to Cs across all reads across all selected cells for the given gene. Figure 8a provides a schematic overview of how the T>C conversion data arises from the 4sU scRNA-seq experimental protocol.

#### ChIP-seq

Publicly available ChIP-seq datasets for ten active HMs produced with K562 cells were downloaded for our analysis. A H3K4me3 ChIP-seq dataset was obtained from the GEO series GSE108323 with sample ID GSM2895356, which had been processed with alignment to the hg19 human genome build [53]. Seven more ChIP-seq datasets, which had also been processed with alignment to the hg19 human genome build, were obtained from the GEO series GSE29611 with sample IDs GSM733778, GSM733651, GSM733653, GSM733656, GSM733675, GSM733692 and GSM733714, corresponding to H3K9ac, H3K4me2, H3K79me2, H3K27ac, H4K20me1, H3K4me1 and H3K36me3, respectively [54]. Two additional ChIP-seq datasets for H3K18ac (aligned to hg19) and H4K16ac (aligned to hg38) were obtained from the series GSE106964 and GSE158736 with sample IDs GSM2862934 and GSM4809274, respectively [55, 56]. The position and read count information from these datasets was used to obtain the single-base resolution coverage values for each HM. These values were associated with their corresponding genes using the information from the comprehensive gene annotation hg19 (or hg38 for H4K16ac) gtf downloaded from Gencode. Analysis of the correlations between bursting parameter estimates and HM coverage at different sections of the gene was carried out by taking the average coverage value for all bases across the specified section (e.g. from 2k bp upstream of the TSS to the TES) for each gene, so a single value is obtained per gene per HM. Metagene plots were produced by averaging the coverage values for each position through/around the gene across all specified genes, similarly to the metagene analysis described in [57].

### Mathematical modelling

In general, we model bursty transcription as a stochastic process closely related to the standard two-state model, as many previous works have [9, 10, 14]. The two-state model has four possible processes of gene activation, gene repression, transcription and degradation, where transcription may only occur with the gene in an active state while degradation acts continuously. This is represented by the following chemical reaction scheme

$$\begin{aligned} G_{off} \xrightarrow {k_{on}} G_{on} \end{aligned}$$

$$\begin{aligned} G_{on} \xrightarrow {k_{off}} G_{off} \end{aligned}$$

$$\begin{aligned} G_{on} \xrightarrow {\beta } G_{on} + RNA \end{aligned}$$

$$\begin{aligned} RNA \xrightarrow {\delta } \emptyset \end{aligned}$$

in which $k_{on}$, $k_{off}$, $\beta$ and $\delta$ represent the rate constants for gene activation, gene repression, transcription and RNA degradation, respectively, while $G_{off}$, $G_{on}$ and *RNA* represent the different species of repressed gene, active gene and transcript, respectively. A schematic representation of the system is shown in Fig. 8b. Gene activation is known to involve facilitated diffusion, in which TFs not only diffuse through the cytoplasm but can also slide along the DNA after becoming associated, to find the target/activation site. This makes the process more complicated than the simple on/off switch of our model, which reflects TF association/disassociation events without DNA sliding. However, previous modelling work has shown that the two-state model accurately captures gene activation due to the high speeds at which DNA-binding proteins slide along the DNA observed in biological systems, which ensures that they do not influence the transcriptional bursting dynamics [58].

With this model, we have burst frequency, $\kappa =\frac{1}{(1/k_{on})+(1/k_{off})}$ and burst size, $b=\frac{\beta }{k_{off}}$, and we recall the burst rate, $a=\frac{\kappa }{\delta }$. Aiming to understand bursting and its timescales specifically, we make the assumption that bursts occur instantaneously, arrive according to a Poisson process and burst in a geometric fashion, which is valid when $\delta<< k_{off}$ since a transcript produced in a given burst is unlikely to have degraded before the burst is over [7, 59], and when $k_{on}<<k_{off}$, which is supported by the parameter estimates reported in [32]. This model simplifies $\kappa =\lim \limits _{k_{off}\rightarrow \infty }\frac{1}{(1/k_{on})+(1/k_{off})}=k_{on}$ while *b* remains finite with $b=\lim \limits _{\beta ,k_{off}\rightarrow \infty }\frac{\beta }{k_{off}}$ [60].

#### Model 1

**Fig. 9:**
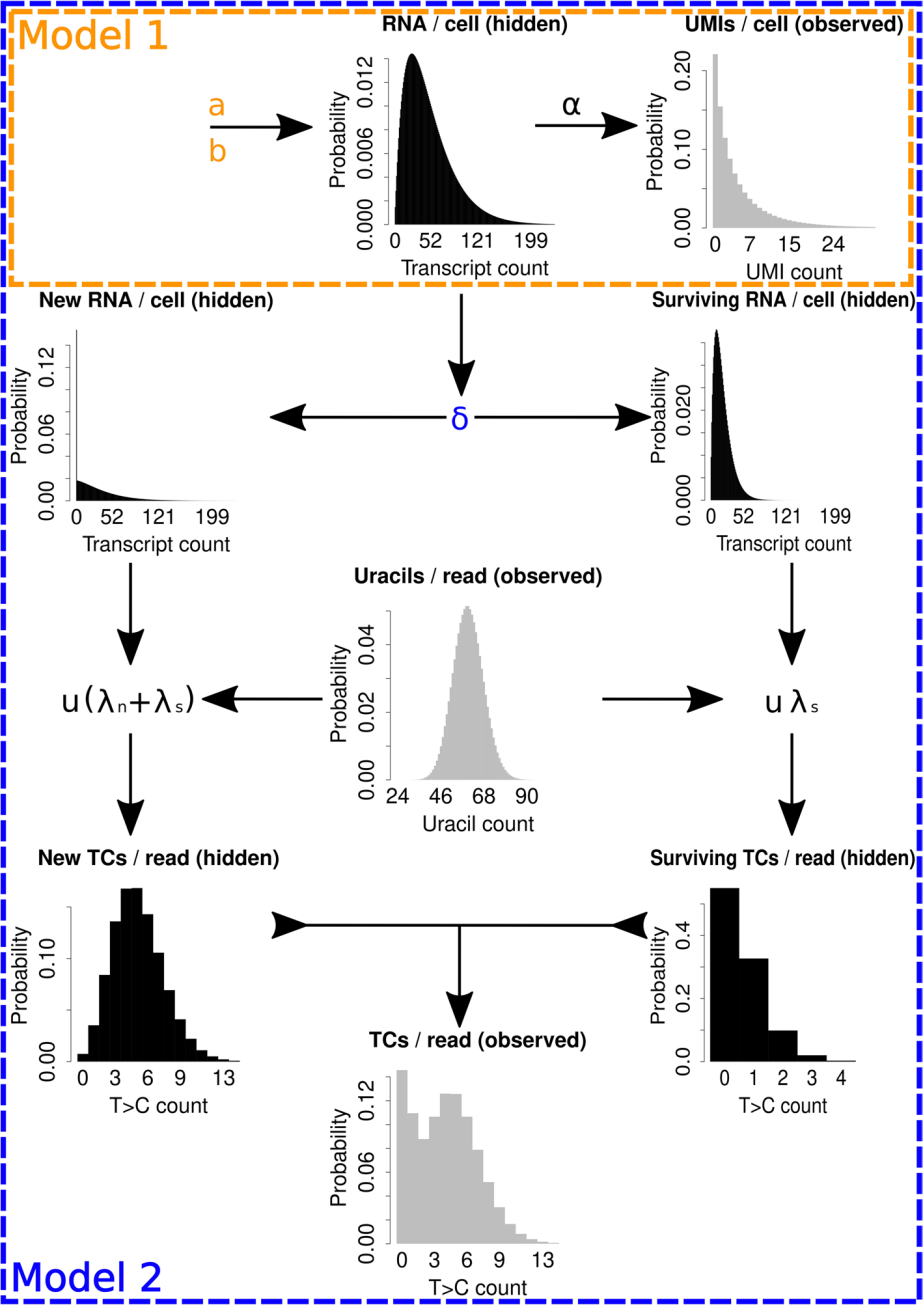
Schematic showing several of the hidden (black) and observed (grey) data we model and their governing parameters. For this illustration, values were set as $a=2$, $b=25$ and $\delta =0.001$ for the biological parameters and $t=1000$, $u\sim Pois(60)$, $\lambda _n=0.075$, $\lambda _s=0.01$ and $\alpha \sim Beta(1,9)$ for the technical parameters. The encompassing boxes indicate the information used during parameter inference by model 1 (*a* and *b*) and 2 (*a*, *b* and $\delta$). The direction of the arrows indicate how the distributions feed into each other as dictated by the accompanying parameters. For example, *a* and *b* determine the steady state distribution, which determines the new and surviving transcript count distribution as dictated by $\delta$ for given *t*, while the new and surviving T>C count distributions combine to form the observed T>C count distribution, which is conditional upon the cell’s transcript count, *m*, with $m=100$ shown here. More information on estimating $\alpha$ and $\lambda _n$ specifically for the Qiu dataset is found in Additional file 1: Figs. S1 and S2, respectively

The first model aims to model the observed unique molecular identifier (UMI) counts of a given cell, *l*, from the estimated capture efficiency (Additional file 1: Fig. S1) of that cell, $\alpha$, in a similar fashion to the technical noise model outlined in [61]. The capture efficiency, $\alpha$, represents the transcript detection rate for that cell (probability of at least one read corresponding to a particular transcript). Based on the instantaneous bursting version of the two-state model described above, the steady state distribution of the transcript count, *m*, can be derived directly from the master equation and corresponds to the negative binomial distribution [10, 59, 60, 62]

1 $$\begin{aligned} P(m)=f_{N\ Bin}\left( m\vert a, \frac{b}{1+b}\right) \end{aligned}$$

which is illustrated by the schematic in Fig. 9, where

$$\begin{aligned} f_{N\ Bin}\left( m\vert a, \frac{b}{1+b}\right) =\frac{\Gamma (m+a)}{\Gamma (m+1)\Gamma (a)}\left( \frac{1}{1+b}\right) ^a\left( \frac{b}{1+b}\right) ^m \end{aligned}$$

The full derivation is available in [60]. We may then model the probability distribution of observing *l* UMIs given *m* transcripts in the cell with a capture efficiency of $\alpha$, as a poisson approximation of the true binomial process

2 $$\begin{aligned} P(l\vert m, \alpha )=f_{Pois}(l\vert m\alpha ) \end{aligned}$$

where

$$\begin{aligned} f_{Pois}(l\vert m\alpha )=\frac{(m\alpha )^le^{-m\alpha }}{l!} \end{aligned}$$

which is valid when $\alpha$ is small. We model the observed data, linked by the unobserved steady state transcript distribution by compounding Eqs. 1 and 2 across the state space of *m* and marginalise

3 $$\begin{aligned} P(l\vert \alpha )=\sum \limits ^{M}_{m=0}P(l\vert m,\alpha )P(m) \end{aligned}$$

where *M* is an upper bound corresponding to the 0.9999 quantile of Eq. 1, which avoids summing to $\infty$, achieving a finite state projection (FSP) [63, 64] with an error of 0.0001. The resulting distribution and its dependence on *P*(*m*) is shown in Fig. 9. The approximation in Eq. 2 permits non-zero probability values when $m<l$, which allows our MCMC algorithm to more efficiently escape regions of parameter space for which $M<l$. This leads us to the likelihood function of model 1 by taking the product of Eq. 3 across all cells in the data

4 $$\begin{aligned} P(L\vert \theta )=\prod _c P(l_c\vert \alpha _c) \end{aligned}$$

where $l_c$ and $\alpha _c$ represent the observed UMI count (for the given gene) and capture efficiency for cell *c*, respectively, and $L=(l_1,\ldots ,l_k)$, with *k* cells in total in the data and $\theta =(\mu ,a,\gamma )$. Since we wish to infer the values of $\theta$ for each gene from the data using this model, we aim to obtain the posterior

5 $$\begin{aligned} P(\theta \vert L)=\frac{P(L\vert \theta )P(\theta )}{\int _{\theta }P(L\vert \theta )P(\theta )d\theta } \end{aligned}$$

which we achieve through MCMC sampling.

#### Model 2

We will now construct a model which unifies the UMI and T>C conversion aspects of the data with the aim of understanding both bursting dynamics and the timescale upon which they occur. Figure 8a illustrates how the T>C conversion data arises from the experimental protocol. First of all, we define $\tau =t\delta$ where *t* is the time before sequencing at which the 4sU nucleotides were added to the cells, otherwise known as the pulse duration. $\tau$ therefore represents unitless time in terms of transcript lifetimes. Next, we must obtain the probability mass function of the number of transcripts surviving to the sequencing point which were produced before the 4sU was added, otherwise known as the surviving transcripts, *s*. This distribution, *P*(*s*), may be understood as the time-decay of the steady state distribution, *P*(*m*), where we have $\lim \limits _{t\rightarrow \infty }P(s=0)=1$ and $P(s\vert t=0)=P(m)$ when $\delta >0$. Degradation acts upon each individual transcript molecule with rate $\delta$, and therefore the probability of a given transcript produced before 4sU was added surviving is $1-F_{Exp}(X \le t \vert \delta )=f_{Pois}(0\vert \tau )$. Therefore, the probability of having *s* transcripts surviving given *m* originally is

6 $$\begin{aligned} P(s\vert m)=f_{Bin}(s\vert m, f_{Pois}(0\vert \tau )) \end{aligned}$$

where

$$\begin{aligned} f_{Bin}(s\vert m, f_{Pois}(0\vert \tau ))={m \atopwithdelims ()s} f_{Pois}(0\vert \tau )^s F_{Exp}(X \le t \vert \delta )^{m-s} \end{aligned}$$

and

$$\begin{aligned} F_{Exp}(X \le t \vert \delta )=1-e^{-\tau } \end{aligned}$$

giving the conditional distribution of *s*. Compounding this with the steady state distribution (Eq. 1) we obtain the marginal

7 $$\begin{aligned} P(s)=\sum \limits _{m=0}^M P(s\vert m)P(m) \end{aligned}$$

We compute this distribution efficiently by using the approximation

8 $$\begin{aligned} P(s)=f_{N\ Bin}\left( m\vert a, \frac{f_{Pois}(0\vert \tau )b}{1+f_{Pois}(0\vert \tau )b}\right) \end{aligned}$$

Next, we obtain the probability mass function of the newly synthesised transcript count, *P*(*n*), for those transcripts that were produced after the 4sU was added and therefore have a higher T>C conversion rate than the background. This may be understood in reverse to *P*(*s*), as it describes the convergence of the newly synthesised transcript count from a point mass at zero to the steady state distribution where we have $P(n=0 \vert t=0)=1$ and $\lim \limits _{t\rightarrow \infty }P(n)=P(m)$ when $a, b, \delta > 0$. An approximate solution to such a distribution was derived as a model of translation in [59] though the assumed relationships apply here. The solution is

9 $$\begin{aligned}{} & {} P(n)= \nonumber \\ \quad{} & {} \frac{\Gamma (a+n)}{\Gamma (n+1)\Gamma (a)}\left( \frac{b}{1+b}\right) ^n\left( \frac{1+be^{-\tau }}{1+b}\right) ^a{}_2F_1\left( -n,-a,1-a-n;\frac{1+b}{e^{\tau }+b}\right) \end{aligned}$$

which is valid when $k_{off}>>\delta$ and $\tau>>\delta /k_{off}$, where ${}_2F_1$ refers to the hypergeometric function. The general dependency of the surviving and new transcript distributions on *P*(*m*), as dictated by $\delta$, is illustrated by Fig. 9. Next, we obtain the probability distribution of transcripts at steady state conditional on our observed cell-specific capture efficiency, $\alpha$, and UMI count, *l*, by using Eqs. 1 and 2

10 $$\begin{aligned} P(m\vert l, \alpha )=\frac{P(l\vert m,\alpha )P(m)}{\sum _m P(l\vert m,\alpha )P(m)} \end{aligned}$$

Now, we describe the probability distribution of *n* conditional on *m* as the joint distribution of *n* and *s*

11 $$\begin{aligned} P(n\vert m)=\frac{P(n)P(s=m-n)}{\sum _{n=0}^m P(n)P(s=m-n)} \end{aligned}$$

with the convolution $\sum _{n=0}^m P(n)P(s=m-n)\approx P(m)$ being used as a normalising value in place of *P*(*m*) due to the approximate nature of *P*(*n*), ensuring that $\sum _{n=0}^mP(n\vert m)=1$. It is now possible to model the number of T>C conversions observed in a given read conditional on *m*, where we have expanded and built upon the poisson mixture model of conversions described in [36] and compounding with Eq. 11

12 $$\begin{aligned}{} & {} P(i\vert m)= \nonumber \\ \quad{} & {} \sum \limits _{n=0}^m\sum \limits _u P(u) \left( \frac{n}{m}f_{Pois}(i\vert u(\lambda _n+\lambda _s))+\left( 1-\frac{n}{m}\right) f_{Pois}(i\vert u\lambda _s)\right) P(n\vert m) \end{aligned}$$

where *P*(*u*) is the gene-specific empirical probability mass function of observing *u* uracils across the fasta sequence corresponding to a given read’s mapping position. $\lambda _s$ is the gene-specific background conversion rate observed in the control dataset (without the addition of 4sU) which represents conversion due to random mutations or other sources outside of chemical conversion. $\lambda _n$ is the gene-invariant conversion rate due to 4sU incorporation and conversion which was estimated from the data (Additional file 1: Fig. S2). *P*(*u*) and the conditional T>C count distribution are shown in Fig. 9, along with the dependence of $P(i\vert m)$ on *P*(*u*), *P*(*s*) and *P*(*n*) as dictated by $\lambda _n$ and $\lambda _s$. We may now model the cell-specific T>C conversion rate for the given gene by compounding Eqs. 10 and 12

13 $$\begin{aligned} P(i\vert l,\alpha )=\sum \limits _{m=0}^M P(i\vert m)P(m\vert l, \alpha ) \end{aligned}$$

where *M* is an upper bound corresponding to the 0.9999 quantile of Eq. 1, again giving a FSP with error 0.0001. We are finally in a position to complete the model and link all our observables together. The observed counts of conversions in each cell may be represented by *y*, where $y_i$ is the number of reads that have *i* conversions. Therefore, the cell-specific observed distribution of conversions per read may be understood as a multinomial distribution with a probability vector determined by Eq. 13

14 $$\begin{aligned} P(y\vert l,\alpha )=\frac{(\sum _i y_i)!}{\prod _i y_i !}\prod _i P(i\vert l,\alpha )^{y_i} \end{aligned}$$

enabling us to model the conversion data conditional on the UMI data. A likelihood function may now be obtained with

15 $$\begin{aligned} P(Y\vert L,\theta )=\prod _c P(y_c\vert l_c,\alpha _c) \end{aligned}$$

where $y_c$ is the conversions per read distribution observed in cell *c* and $Y=(y_1,\ldots ,y_k)$ where $y_{c,i}$ is the number of reads with *i* conversions in cell *c* for the given gene. The final likelihood function of model 2 is now defined as the product of Eqs. 4 and 15

16 $$\begin{aligned} P(Y,L\vert \theta )=P(Y\vert L,\theta )P(L\vert \theta ) \end{aligned}$$

As in Eq. 5, MCMC sampling was used to obtain

17 $$\begin{aligned} P(\theta \vert Y,L)=\frac{P(Y,L\vert \theta )P(\theta )}{\int _{\theta }P(Y,L\vert \theta )P(\theta )d\theta } \end{aligned}$$

One thing to note about model 2 is that Eq. 9 is an approximate solution and breaks down in certain regions of parameter space. When *a* and/or *b* become too large and/or $\tau$ becomes too small, the function will oscillate around the true probability distribution function, with these oscillations quickly becoming more extreme to the point that the approximate solution gives negative probability values. The solution can be said to become unstable in these regions of parameter space, and therefore such regions will be referred to as unstable parameter space. If a gene is found to reside within an unstable region of parameter space then an alternative to model 2 must be used.

#### Model 3

Our final model acts as an alternative to model 2 when a gene resides within an unstable region of parameter space. Unlike model 2, this model ignores the cell-specific T>C information in favour of simply pooling the conversions across all cells. We define the probability distribution of observing *i* conversions for a given read

18 $$\begin{aligned} P(i)=\sum \limits _u P(u) \left[ F_{Exp}(X \le t \vert \delta )f_{Pois}(i\vert u(\lambda _n+\lambda _s))+f_{Pois}(0\vert \tau )f_{Pois}(i\vert u\lambda _s)\right] \end{aligned}$$

This is similar to Eq. 12 but is independent of the total transcript count, *m*, and is therefore not cell specific. We can apply Eq. 18 to the full set of observed conversions across cells, *Y*, again using the multinomial distribution to obtain a likelihood function

19 $$\begin{aligned} P(Y\vert \theta )=\frac{(\sum _i y_i)!}{\prod _i y_i !}\prod _i P(i)^{y_i} \end{aligned}$$

where $y_i$ represents the number of reads with *i* conversions summed across all cells rather than being a cell-specific value as in Eqs. 14 and 15. We define the final likelihood function of model 3 as the product of Eqs. 4 and 19.

20 $$\begin{aligned} P(L,Y\vert \theta )=P(L\vert \theta )P(Y\vert \theta ) \end{aligned}$$

As in Eqs. 5 and 17, MCMC sampling was used to obtain

21 $$\begin{aligned} P(\theta \vert L,Y)=\frac{P(L,Y\vert \theta )P(\theta )}{\int _{\theta }P(L,Y\vert \theta )P(\theta )d\theta } \end{aligned}$$

### Markov chain Monte Carlo algorithm

MCMC was employed in order to sample from the posterior distributions outlined in Eqs. 5, 17 or 21 using a Metropolis-adjusted Langevin algorithm (MALA) within a Gibbs sampler, which simulates a Markov chain using Langevin dynamics [65] and corrects the Euler-Maruyama integration error with an accept-reject step as with the Metropolis-Hastings algorithm [66]. The chain is initialised semi-randomly, setting $\theta ^{(1)}$ in a manner which takes advantage of the information immediately available from the data to start the chain relatively close to the target density. We calculate empirical estimates of the expression level, $\mu$, and transcript lifetime, $\gamma$, as

$$\begin{aligned} \hat{\mu }=\frac{1}{N}\sum \limits _{c=1}^{N}l_c/\alpha _c \end{aligned}$$

where $N=795$ is the number of cells in the dataset, and as

$$\begin{aligned} \hat{\gamma }=-t/\log (\max [0.1,\min \{0.9,(1-((\lambda -\lambda _s)/\lambda _n)\}]) \end{aligned}$$

where $\lambda$ is the observed conversion rate for the given gene across all reads, while $\lambda _s$ and $\lambda _n$ represent the background conversion rate measured in the control dataset and the estimated 4sU-mediated conversion rate, respectively. We then set $\mu =\hat{\mu }$ and draw

$$\begin{aligned} a\sim LUnif(1,10) \end{aligned}$$

and

$$\begin{aligned} \gamma \sim \mathcal {N}(\hat{\gamma },\hat{\gamma } / 5) \end{aligned}$$

where

$$\begin{aligned} f_{LUnif}(x\vert y,z)=\frac{1}{x\ln (z/y)} \end{aligned}$$

with support [*y*, *z*] for $y>0$ and

$$\begin{aligned} f_{\mathcal {N}}(x\vert M,\sigma )=\frac{1}{\sigma \sqrt{2\pi }}e^{-\frac{1}{2}\left( \frac{x-M}{\sigma }\right) ^2} \end{aligned}$$

We repeatedly draw $\theta ^{(1)}$ in this way until $P(X\vert \theta ^{(1)})P(\theta ^{(1)})>0$ where *X* is the dataset and $P(\theta )$ represents the prior distribution, which in this case is defined to be an uninformative multivariate uniform distribution such that

$$\begin{aligned} P(\theta =(\mu ,a,\gamma ))=f_{Unif}(\mu \vert 0,100000)f_{Unif}(a\vert 0,100000)f_{Unif}(\gamma \vert 1,100000) \end{aligned}$$

where

$$\begin{aligned} f_{Unif}(x\vert y,z)=\frac{1}{z-y} \end{aligned}$$

with support [*y*, *z*]. At each step, *j*, in the Markov chain, the next step is sampled by proposing jumps to new positions in parameter space from the current position, proceeding through three dimensional parameter space with $\theta =(\mu ,a,\gamma )$. This parameterisation was chosen for Markov chain progression to minimise correlations between parameters and proposals to negative (unsupported) values. The classic Metropolis-Hastings algorithm [66] corresponds to a random walk through parameter space, which converges relatively slowly to the target density, and which samples from the posterior inefficiently due to slow mixing of the chain, with the optimal acceptance rate (proportion of accepted proposals) being only 0.234 [67]. Therefore, we make use of the MALA as a superior alternative, which converges much more efficiently, requiring only $O(d^{1/3})$ steps, where *d* is the dimension of the target density, whereas the random walk requires *O*(*d*) steps, while the higher optimal acceptance rate of 0.574 allows for faster mixing and reduced dependence between samples [65]. The Markov chain is treated as an itô diffusion and behaves according to Langevin dynamics with stochastic differential equation

22 $$\begin{aligned} d\theta _t=\nabla \log \pi (\theta _t)+\sqrt{2}dW_t \end{aligned}$$

evolving $\theta$ in imaginary time with a standard Brownian motion diffusion term, *W*, and a drift term determined by the vector gradient, $\nabla$, of the logarithm of the posterior density, $\pi (\theta )\propto P(X\vert \theta )P(\theta )$, with respect to $\theta$ evaluated at $\theta _t$. However, we do not have an analytical solution for $\nabla \log \pi (\theta )$ which means we must estimate this numerically using the change in likelihood observed between the current step, *j*, and the previous one when generating a proposal. This leads to an additional complication, wherein we may not propose a new sample for all parameters simultaneously since then the observed change in likelihood would be the combined effect of the change in each parameter, making the individual gradients impossible to estimate. Therefore, we must sequentially update each parameter conditional on the current value of all other parameters, which are treated as fixed constants. This corresponds to embedding our MALA within a Gibbs sampler [68, 69], meaning that *d* sub-steps are required to move from step *j* to $j+1$. At step *j*, we cycle through each parameter, *k*, from 1 to *d*, and draw a new proposal for parameter *k* from a proposal distribution as determined by Eq. 22

$$\begin{aligned} \theta _k^{(*)}=\theta _k^{(j)}+S_k\nabla _k\log \pi (\theta )+\sqrt{2S_k}\xi \end{aligned}$$

where $\xi$ is a standard normal random variable and *S* is an adaptive scaling constant such that the proposal is drawn from

$$\begin{aligned} \theta _k^{(*)}\sim \mathcal {N}\left( \theta _k^{(j)}+S_k\nabla _k\log \pi (\theta ),\sqrt{2S_k}\right) \end{aligned}$$

This is accepted with a probability given by the likelihood ratio at the proposed and current value

23 $$\begin{aligned} A=\min \left( 1,\frac{\pi (\theta _{1}^{(j+1)},\ldots ,\theta _{k-1}^{(j+1)},\theta _{k}^{(*)},\theta _{k+1}^{(j)},\ldots ,\theta _{d}^{(j)})}{\pi (\theta _{1}^{(j+1)},\ldots ,\theta _{k-1}^{(j+1)},\theta _{k}^{(j)},\ldots ,\theta _{d}^{(j)})} \right) \end{aligned}$$

where substituting $\pi (\theta )$ for $P(X\vert \theta )P(\theta )$ gives an equivalent ratio due to the proportionality, which allows us to refer directly to the target density, $\pi$. Note that the intractable integrals in the denominators of Eqs. 5, 17 and 21 cancel out to allow the acceptance probability to be calculated with only the likelihood function and the prior density. In our special case with uniform priors, these also cancel, only serving to reject proposals outside of the plausible ranges of parameter space as defined by the prior. With probability *A* we set $\theta _k^{(j+1)}=\theta _k^{(*)}$, otherwise $\theta _k^{(j+1)}=\theta _k^{(j)}$ and since we treat parameters other than $\theta _k$ as constants, we iteratively draw $\theta$ from the conditional rather than joint densities as

$$\begin{aligned} \theta _{k}^{(j+1)}\sim P(\theta _{k}^{(j+1)}\vert \theta _{1}^{(j+1)},\ldots ,\theta _{k-1}^{(j+1)},\theta _{k+1}^{(j)},\ldots ,\theta _{d}^{(j)}) \end{aligned}$$

If the proposal is accepted, we update our estimate of the local gradient for the parameter *k* as

$$\begin{aligned} \nabla _k=\frac{\log \pi (\theta _k^{(j+1)})-\log \pi (\theta _k^{(j)})}{\theta _k^{(j+1)}-\theta _k^{(j)}} \end{aligned}$$

otherwise we set $\nabla _k=0$. We also recursively update the adaptive scaling constant associated with parameter *k* in the manner described for the Adaptive Scaling Metropolis algorithm of [67]

$$\begin{aligned} S_k=e^{(\log (S_k)+\eta (A-0.574))} \end{aligned}$$

with a recursively updated decay term

$$\begin{aligned} \eta =0.999\eta \end{aligned}$$

which in the long-term results in the MALA mixing close to the optimal parameter-specific acceptance rate of 0.574 [65]. At step 1, we initialise $\nabla =0$, $S=\theta ^{(1)}/100$ and $\eta =0.1$. Density plots indicate that in the vast majority of cases acceptance rates close to 0.574 were achieved (Additional file 1: Fig. S5).

The process repeats until 5000 steps have been completed ($j=5000$) if $\hat{\mu }<1000$ or 1500 if $\hat{\mu }\ge 1000$, since for these genes with very high expression levels each step takes longer but the stronger evidence means that fewer steps are required. Therefore, the Markov chain converges to the posterior distribution according to its gradient. Posteriors were produced from the sampled chain using the last 1000 or 2500 steps for high expression or other genes, respectively, with a thinning factor of 2, where only every 2nd point in the chain is used in order to reduce dependency between points, resulting in smoother posterior densities and sample sizes of 500 or 1250. When using model 2, for each step, we check if the proposal for any sub-step was rejected because of negative probability values appearing in Eq. 9 due to the approximate non-equilibrium solution failing for an unstable point in parameter space. We set a rolling window size, *w*, equal to 100 or 500 for high expression or other genes, respectively. We then check at each step, *j*, if the number of steps with a rejection of this nature is $\ge w/20$ for steps $[\max ((w/2)+1,j-w+1),j]$ and if this condition is met then the Markov chain is restarted using model 3 instead of model 2.

### Simulations for model comparison

The performance of inference using different likelihood functions was tested on simulated data. Gillespie’s exact algorithm (stochastic simulation algorithm) [70] was used to simulate data according to the reactant matrix shown in Table 3 and the product matrix shown in Table 4, with the stoichiometry matrix shown in Table 5.

**Table 3:** Reactant matrix for new and surviving transcript count Gillespie algorithm simulations

	$RNA_0$	$RNA_1$	$G_{on}$	$G_{off}$
$\beta _0$	0	0	1	0
$\beta _1$	0	0	1	0
$\delta _0$	1	0	0	0
$\delta _1$	0	1	0	0
$k_{on}$	0	0	0	1
$k_{off}$	0	0	1	0

**Table 4:** Product matrix for new and surviving transcript count Gillespie algorithm simulations

	$RNA_0$	$RNA_1$	$G_{on}$	$G_{off}$
$\beta _0$	1	0	1	0
$\beta _1$	0	1	1	0
$\delta _0$	0	0	0	0
$\delta _1$	0	0	0	0
$k_{on}$	0	0	1	0
$k_{off}$	0	0	0	1

**Table 5:** Stoichiometry matrix for new and surviving transcript count Gillespie algorithm simulations

	$RNA_0$	$RNA_1$	$G_{on}$	$G_{off}$
$\beta _0$	1	0	0	0
$\beta _1$	0	1	0	0
$\delta _0$	-1	0	0	0
$\delta _1$	0	-1	0	0
$k_{on}$	0	0	1	-1
$k_{off}$	0	0	-1	1

This allows simulation of the pool of transcripts in the cell that was synthesised before ($RNA_0$) and after ($RNA_1$) the 4sU pulse started. The simulation is run with initial conditions $X_0=(0,0,0,1)$ where $X=(RNA_0,RNA_1,G_{on},G_{off})$ and rate constant values are set for bursty expression $\theta =(\beta _0=50,\beta _1=0,\delta _0=0.001,\delta _1=0.001,k_{on}=0.0005,k_{off}=1)$, running until $t_0=200000$ to bring the system to steady state. The system state at the end of this run, $X_{t_0}$, is then used as the initial condition for a second run, where we now set $\theta =(\beta _0=0,\beta _1=50,\delta _0=0.001,\delta _1=0.001,k_{on}=0.0005,k_{off}=1)$ to simulate the newly synthesised transcripts produced during the 4sU pulse along with decay of pre-existing transcripts. A pulse duration of $t_1=1000$ min was used here, giving the final state of the system $X_{t_1}$, and importantly giving the counts for $RNA_0$ and $RNA_1$ in the cell. This was repeated to simulate $N=10000$ cells. In-silico sequencing data was then generated based on these simulated transcript count values. Cell-specific capture efficiencies were drawn

$$\begin{aligned} \alpha \sim Beta(1,9) \end{aligned}$$

before drawing the cell-specific UMI counts, *l*, corresponding to the two pools of transcripts as

$$\begin{aligned} l_k\sim Bin(RNA_k,\alpha ) \end{aligned}$$

for $k=0$ and $k=1$, so that the total UMI count for the given cell is $l=l_0+l_1$. The cell-specific total number of reads corresponding to each UMI in the two pools is then drawn

$$\begin{aligned} r_{k,j}\sim ZTPois(\nu ) \end{aligned}$$

where $\nu =5$ represents sequencing depth and reads per UMI is a zero-truncated Poisson random variable with

$$\begin{aligned} f_{ZTPois}(r\vert \nu ,r>0)=\frac{\nu ^r}{(e^{\nu }-1)r!} \end{aligned}$$

using the same logic of Poisson assignment of reads to UMIs as in [71]. Then, the cell-specific total number of reads of the given pool is

$$\begin{aligned} r_k=\sum \limits _{j=1}^{l_k}r_{k,j} \end{aligned}$$

The number of uracils across the sequenced part of the transcript is then drawn for each read

$$\begin{aligned} u_{k,j}\sim Pois(\hat{u}) \end{aligned}$$

where $\hat{u}=60$ is the average number of uracils per read. The number of conversions in each read in the cell is then drawn for the two pools of transcripts as

$$\begin{aligned} i_{0,j}\sim Bin(u_{0,j},\lambda _s) \end{aligned}$$

and

$$\begin{aligned} i_{1,j}\sim Bin(u_{1,j},\lambda _s+\lambda _n) \end{aligned}$$

where we set $\lambda _s=0.01$ and $\lambda _n=0.075$. The overall conversion data across all reads in the cell is then $i=(i_0,i_1)$, where $i_0=(i_{0,1},\ldots ,i_{0,r_0})$ and $i_1=(i_{1,1},\ldots ,i_{1,r_1})$, from which we obtain *y*, where $y_i$ is the number of reads with *i* conversions in the given cell. Now we have our simulated dataset which we can use to demonstrate our capacity to recover known parameter values. MCMC was carried out with different likelihood functions in the previously described manner to sample posterior distributions.

## Supplementary information

**Additional file 1.** Supplementary information: Contains methodological information on estimating capture efficiencies and 4sU-mediated TC rates and for simulating data for validating algorithm performance. Also contains results of metagene and correlation analyses for various HMs not shown in the main text, as well as a correlation between our estimated transcript decay rates and previously published cell-matched decay rates.

**Additional file 2.** High vs low noise cell-specific T>C rate distribution transition: Video gif showing the differential transition from surviving to new transcript pool for high and low noise genes through the cell-specific T>C rate distributions for data simulated with different pulse durations.

**Additional file 3.** Review history.

## Data Availability

The inference algorithm has been made available as a GitHub-distributed R package (https://github.com/hebenstreitLab/burstMCMC) [72]. The pre-processing scripts are similarly available in a GitHub repository (https://github.com/hebenstreitLab/burstMCMCpreprocessing) [73]. Both repositories are licensed under the MIT license. The scripts along with the processed data used in the analysis are available in Zenodo (https://doi.org/10.5281/zenodo.7707970) under the MIT license [74]. The raw 4sU scRNA-seq data used can be found under the GEO sample IDs GSM4512696 and GSM4512697 [38, 75]. The processed scRNA-seq dataset used for calculating capture efficiencies can be found under the GEO sample ID GSM1599501 [76, 77]. The processed ChIP-seq datasets used for metagene analysis can be found under GEO sample IDs GSM2895356, GSM733651, GSM733653, GSM733656, GSM733675, GSM733692 GSM733714, GSM733778, GSM2862934 and GSM4809274 [53–56, 78–81].
