# Supplementary material for: Synergising single-cell resolution and 4sU labelling boosts inference of transcriptional bursting
Source: Genome Biol. 2023 Jun 16;24:138. doi: 10.1186/s13059-023-02977-y (PMC10276402; doi:10.1186/s13059-023-02977-y)
Supplement: Supplementary file 1 — Additional file 1. Supplementary information: Contains methodological information on estimating capture efficiencies and 4sU-mediated TC rates and for simulating data for validating algorithm performance. Also contains results of metagene and correlation analyses for various HMs not shown in the main text, as well as a correlation between our estimated transcript decay rates and previously published cell-matched decay rates. [file 13059_2023_2977_MOESM1_ESM.pdf]

Synergising single-cell resolution and 4sU labelling boosts inference of transcriptional bursting

David M Edwards, Philip Davies and Daniel Hebenstreit

Supplementary information

Cell-specific capture efficiencies

Our models require the capture efficiency, α, (proportion of transcripts from each cell with at least 1 corresponding read) of each cell to be known. This necessitates the use of RNA spike-in probes, in which a known quantity of material is added to each cell and the proportion of molecules detected in the sequencing gives α. Spike-ins were not used in the Qiu datasets, but capture efficiencies may be inferred by using data from Klein et al 2015 [1], which has cell-matched (K562) scRNA-seq data (with GEO sample ID GSM1599501) that does contain ERCC spike-in probes. We construct a mathematical model to obtain the probability distribution of the capture efficiencies in the 4sU Qiu dataset, α_q, based on the Klein data, under the assumption that since both datasets were produced with K562 cells, the underlying probability distribution of the total transcript count in each cell, m, is the same for both datasets. According to [2], the true number of spike-in molecules loaded to each cell, x, may be modelled as a poisson random variable with rate based on the expected number of molecules

loaded per cell, λ, (12467.64 in the case of the Klein dataset)

$$x \sim Pois(\lambda)$$

The capture efficiency of each of the 953 cells in the Klein dataset, α_k, is then

$$\alpha_k \sim Beta(y, x - y)$$

where y represents the total number of spike-in molecules detected in the given cell. The total number of transcripts present in each cell in Klein is modelled as

$$m - l_k \sim NBin(l_k, 1 - \alpha_k)$$

where l_k represents the total UMI counts across all genes in Klein for the given cell, using the negative binomial parametrisation defined for equation 1 (Methods). The capture efficiency of each cell in Qiu, α_q, may then be obtained using the total number of UMIs in the given cell across all genes, l_q,

$$\alpha_q \sim Beta(l_q, m - l_q)$$

The probability density function for each cell in Qiu is then solved by numerically integrating the above distributions through random number generation to obtain

$$P(\alpha_q|\lambda, y, l_k, l_q) = \\ \sum_{m=l_q}^{\infty} f_{Beta}(\alpha_q|l_q, m - l_q) \frac{1}{N} \sum_{i=1}^{N} \int f_{NBin}(m - l_{k,i}|l_{k,i}, 1 - \alpha_{k,i}) \\ \sum_{x=y_i}^{\infty} [f_{Beta}(\alpha_{k,i}|y_i, x - y_i) f_{Pois}(x|\lambda)] \, d\alpha_{k,i}$$

where N is the number of cells in Klein, i refers to the ith cell of Klein and

$$f_{Beta}(\alpha_q|l_q, m - l_q) = \frac{\alpha_q^{l_q}(1 - \alpha_q)^{m - l_q}}{B(l_q, m - l_q)}$$

with

$$B(l_q, m - l_q) = \frac{\Gamma(l_q)\Gamma(m - l_q)}{\Gamma(m)}$$

Estimates may be derived from $P(\alpha_q|\lambda, y, l_k, l_q)$ with $E[\alpha_q]$ and confidence may be quantified with $E[\alpha_q^2]$. Figure S1 indicates high confidence in our estimates through the low CV, while the estimated capture efficiencies for Qiu are lower than for Klein, at around 0.02 on average. A quick, simple method for calculating α_q estimates without quantifying confidence is as follows

$$\alpha_q = l_q/\hat{m}$$

where

$$\hat{m} = \frac{1}{N}\sum_{i=1}^{N} l_{k,i}\lambda/y_i$$

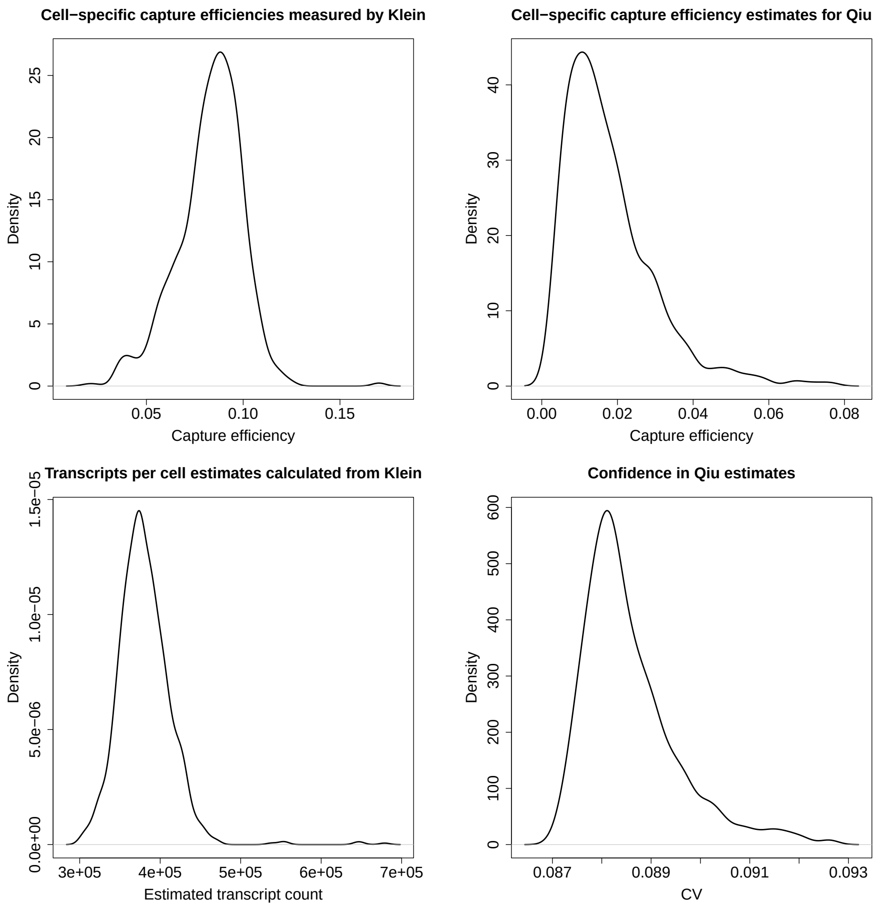

Figure S1: Density plots of the capture efficiencies measured for the Klein dataset, the total transcript content per cell estimated from Klein, the capture efficiencies estimated for the Qiu dataset and the confidence in those estimates as represented by the CVs.

Conversion rates

Models 2 and 3 also require the gene-specific background and gene-invariant 4sU-mediated T>C conversion rates to be known, λ_s and λ_n, respectively. As previously mentioned, λ_s is defined as the proportion of genomic Ts in all reads and all cells that appeared as Cs in the control dataset for the given gene. Therefore, the conversion rate observed in the 4sU dataset corresponds to $\lambda_s +$

λ_n. The conversion rates of all genes for which we have high confidence in the rate estimate in both datasets are shown in figure S2, which was 6259 genes. Confidence is obtained by modelling the T>C rate, λ, as

$$\lambda \sim Beta(C, T - C)$$

classing those with a resulting CV $< 10^{-0.5}$ in both datasets as having high confidence. We expect the rate in the 4sU dataset to be at least as large as in the control, hence genes tend to appear on the diagonal or above it. Genes with higher turnover are expected to appear further above the diagonal while those with low turnover are expected to appear closer to it. The curve along the top of the plot represents λ_s (x-axis) added to our estimate of λ_n. λ_n is estimated by first assuming that all reads correspond to new transcripts (synthesised during the pulse) and then calculating

$$p = 1 - F_{Bin}(C - 1|T, \lambda_s + \lambda_n) = F_{Bin}(T - C|T, 1 - \lambda_s - \lambda_n)$$

for each gene so that $\mathbf{p} = (p_1, \ldots, p_{6259})$ where

$$F_{Bin}(C|T, \lambda_s + \lambda_n) = \sum_{i=0}^{\lfloor C \rfloor} \binom{T}{i} (\lambda_s + \lambda_n)^i (1 - \lambda_s - \lambda_n)^{T-i}$$

The estimate for λ_n is then the minimum value for which $\sum_g [p_g < 10/6259] < 10$. With this approach, $\lambda_n \approx 0.07547$. Estimating λ_n in this manner relies on an assumption that at least the top ~ 10 genes in terms of background-subtracted T>C rate have almost complete turnover of their transcriptomes across cells. Genes with lower T>C rates do not contribute to the estimate because the lower the T>C rate the higher the probability value associated with the binomial survival function, meaning that our new read assumption

does not actually depend upon them. The validity of this assumption is highly probable given the 4 hour long 4sU pulse duration, and is supported by the shape of figure S2. The cloud of points is truncated at the top where the upper bound is, being flat with a high density of points there. If no / very few genes had complete turnover then we would expect the points to thin out towards the top in a manner reflecting the bottom of the cloud, rather than the sharp truncation which is observed. This high density flat top indicates that a rather large number of genes have achieved their maximum T>C rate.

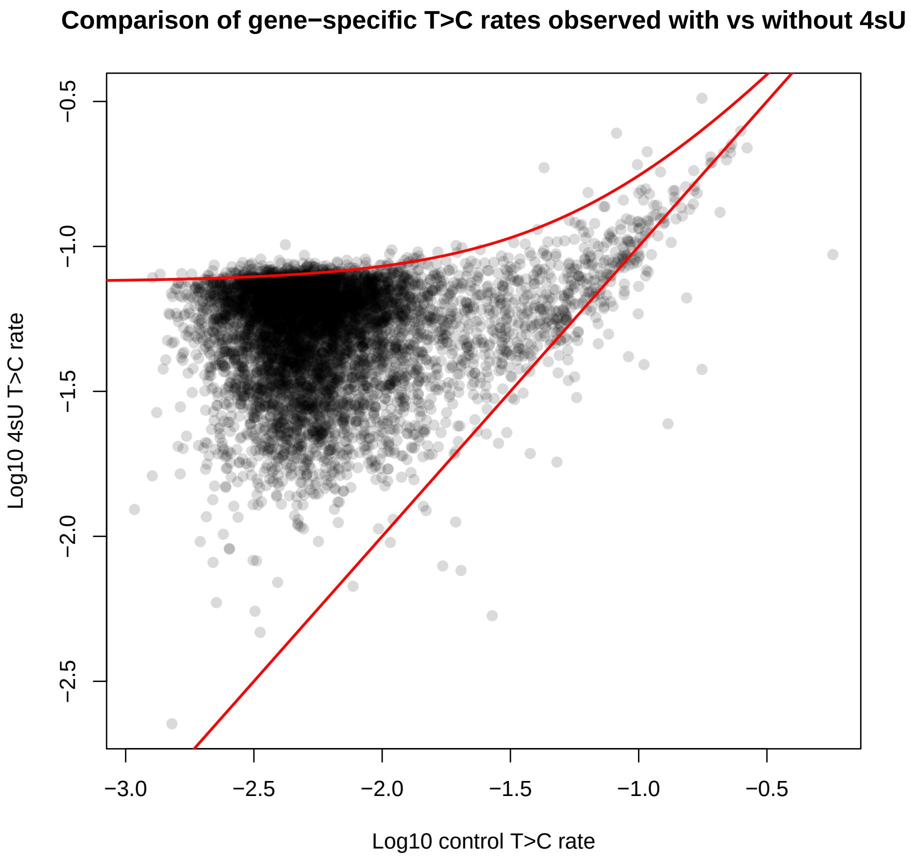

Figure S2: Gene-specific T>C conversion rates in the control vs 4sU datasets for 6259 genes for which we have high confidence in the observed T>C rate in both datasets. The lower red line represents λ_s, while the upper one represents $\lambda_s + \lambda_n$.

Decay rate correlation

Additional confidence in our results is provided by showing the strong correlation between the decay rate estimates we obtained from Qiu for our high confidence genes and those previously calculated in Schofield et al 2018 [3] for the same cell type (figure S3).

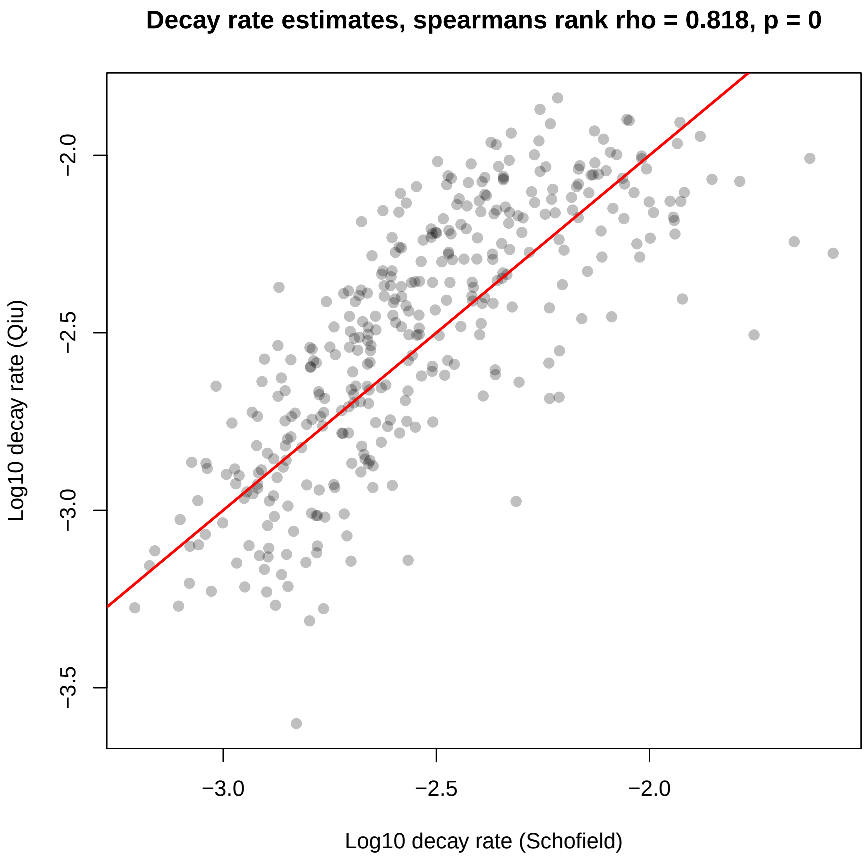

Figure S3: Decay rate (δ) estimates we obtained from Qiu for our high confidence gene set vs those calculated by Scofield for the same genes, with the Spearman's rank correlation statistics shown and the diagonal displayed (red line).

Inference on simulated data

In order to assess the performance of the inference algorithm, a dataset was simulated for each of the 12276 genes from the real Qiu dataset whose bursting dynamics were inferred. The θ estimates obtained from Qiu were used as the "ground truth" parameter values to simulate new datasets. A dataset for each gene is simulated by drawing the steady state transcript counts for $N = 795$

cells, matching the real data, at the start of the pulse

$$m_0 \sim NBin\left(a, \frac{b}{1+b}\right),$$

the surviving transcript counts at the end of the pulse

$$s \sim Bin\left(m_0, e^{-\tau}\right),$$

the number of bursts occurring during the pulse

$$\beta \sim Pois(\kappa t),$$

how long before the end of the pulse each burst occurs

$$T \sim Unif(0, 240),$$

the size of each burst

$$\sigma \sim Geom\left(\frac{1}{1+b}\right)$$

where

$$f_{Geom}\left(\sigma | \frac{1}{1+b}\right) = \left(\frac{b}{1+b}\right)^{\sigma}\left(\frac{1}{1+b}\right)$$

and the number of newly synthesised transcripts from each burst which survive to the end of the pulse

$$n \sim Bin\left(\sigma, e^{-\delta T}\right).$$

Then the total transcripts in each simulated cell at the end of the pulse is $m = s + \hat{n}$, summing the number of transcripts surviving from each burst occurring during the pulse as $\hat{n} = \sum n$. The number of UMIs corresponding to surviving

and new transcripts in each cell is then drawn as

$$l_s \sim Bin(s, \alpha)$$

and

$$l_n \sim Bin(\hat{n}, \alpha)$$

where α is sampled without replacement from the set of estimated capture efficiencies. Then we have the total UMI count for each cell $l = l_s + l_n$ and L, where L_c is the UMI count of cell c. For each UMI, we draw the number of corresponding reads, r, as

$$r \sim ZTPois(\rho)$$

where ρ is the maximum likelihood estimate given the observed ratio of reads to UMIs, R, across all cells for the given gene in the real data. ρ is obtained by minimising

$$\left| R - \frac{\rho}{1 - e^{-\rho}} \right|$$

From this we have the total number of reads corresponding to surviving, r_s, and new, r_n, transcripts for each cell. The number of uracils, u, in each read is then drawn from the gene-specific empirical probability mass function, $P(u)$, from equation 12 (Methods). Finally, the number of uracils in each read which undergo a T>C conversion, i, is then drawn for surviving reads as

$$i \sim Bin(u, \lambda_s)$$

and for new reads as

$$i \sim Bin(u, \lambda_s + \lambda_n)$$

Now we have y, where y_i represents the number of reads with i total conversions in the given cell and Y, where Y_c is the vector y for cell c. We may now carry out inference with L and Y as previously described using model 2 (or alternatively model 3, Methods). The same selection of genes that was used for the real data was applied, selecting based on a maximum CV of 0.45 across all parameters. The correlations between the ground truth values and the parameter estimates derived from our sampled posteriors for the 422 selected simulated genes are shown in figure S4. The strong, tight correlations about the diagonal demonstrate the successful recovery of ground truths for all parameters. The error increases for genes with very low b or δ, which reflects the increased CV for such estimates shown in figure 4. Higher error for a estimates primarily corresponds to those genes with very low, error-prone b values.

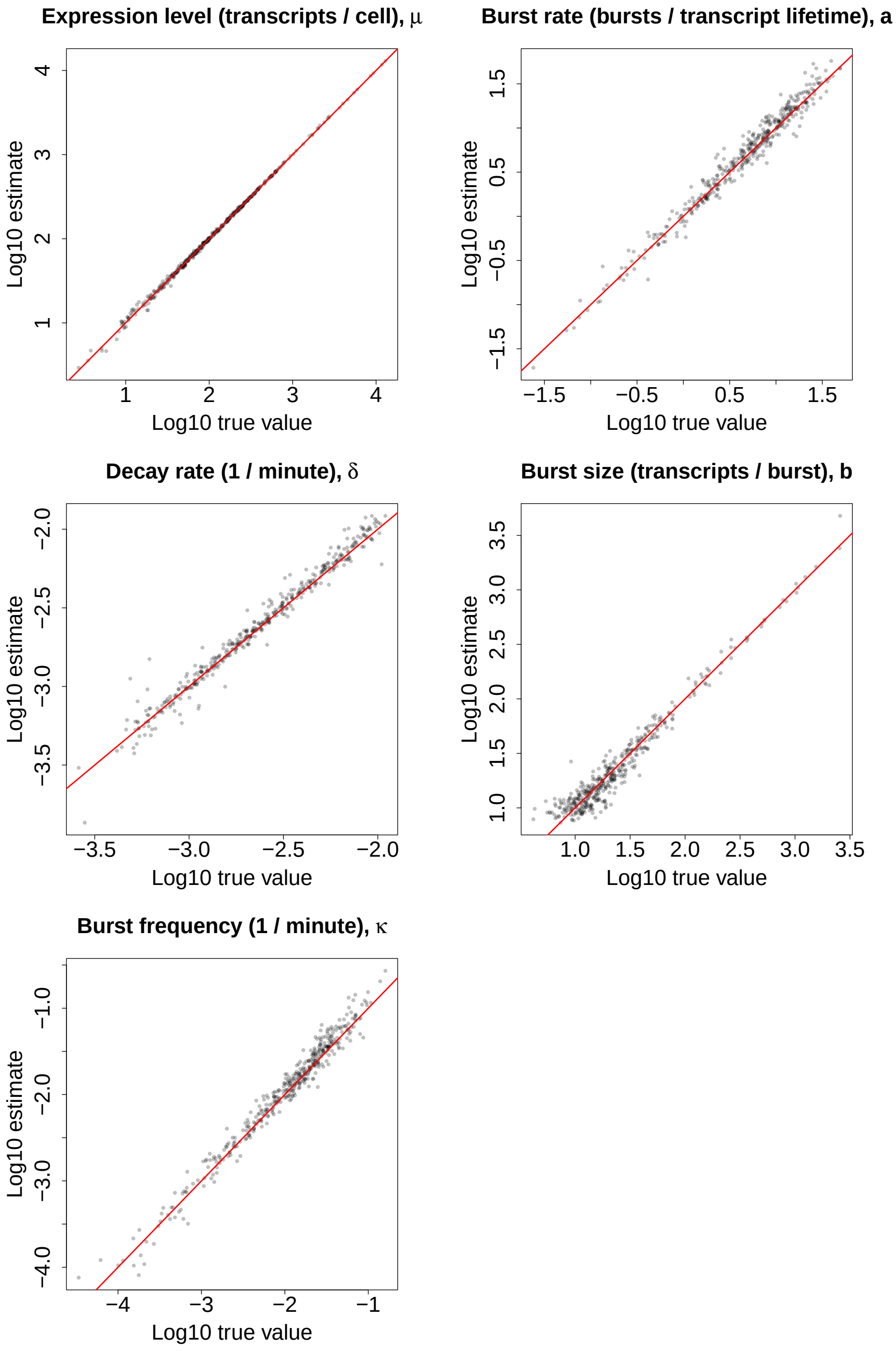

Figure S4: Correlations between true parameter values and those estimated from sampled posteriors for 422 selected simulated genes, with red lines representing diagonals.

Acceptance rates

Density plots of the MCMC acceptance rates (after burn-in removal) for all 12276 genes analysed in the Qiu dataset and the corresponding simulated genes are shown (figure S5). This diagnostic indicates that overall the desired mixing behaviour is achieved in all cases for each of the three parameters in our chosen parametrisation, with the ideal acceptance rate being roughly 0.574 [4].

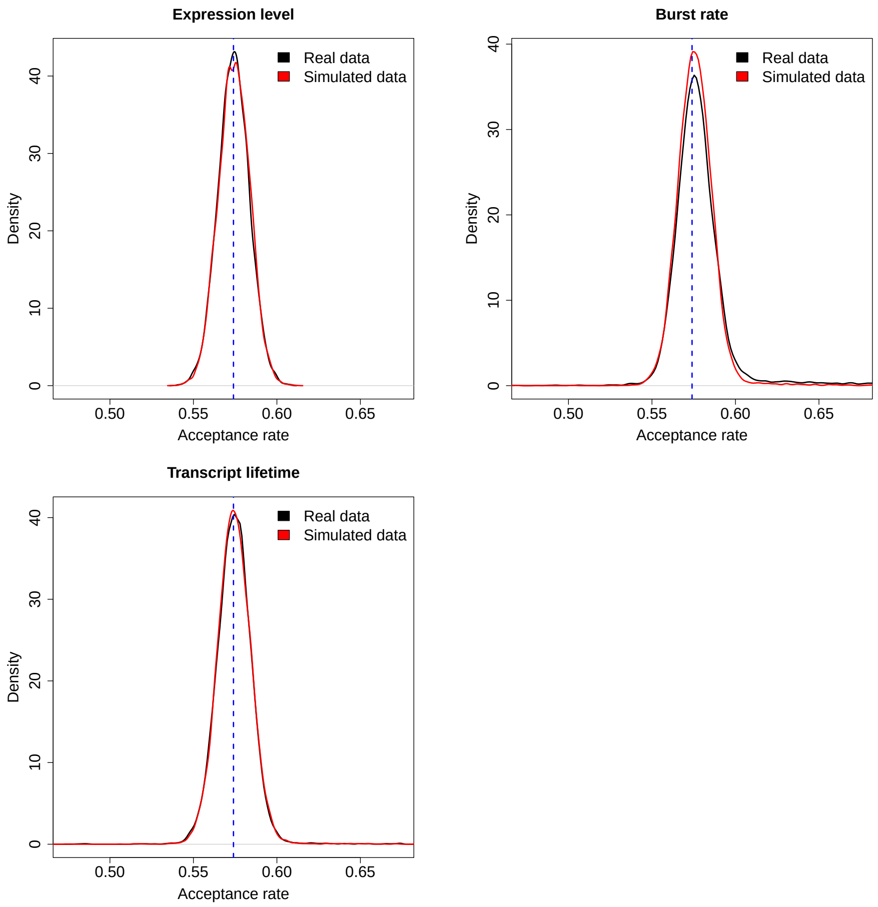

Figure S5: Markov chain acceptance rates (excluding burn-in) for inference on the Qiu dataset and the corresponding simulated dataset for each of the three parameters in our chosen parametrisation, with the vertical dashed lines indicating the optimal acceptance rate (0.574).

Further metagene and correlation analyses

Promoter-localised histone modifications

Metagene analyses of the other promoter-localised HMs that were represented by H3K4me2 (Results) shows the profiles for H3K4me3 (figure S6), H3K9ac (figure S7) and H3K27ac (figure S8).

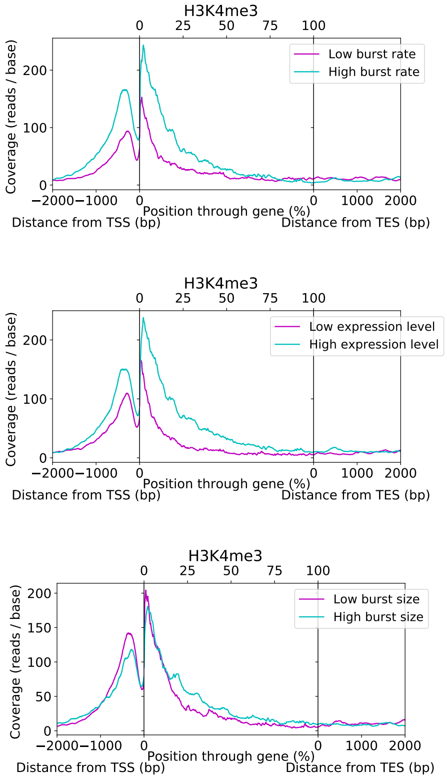

Figure S6: Metagene plots of H3K4me3 coverage, comparing profiles for the top and bottom 50% of genes when split according to their estimates for each parameter, denoted by high and low, as indicated.

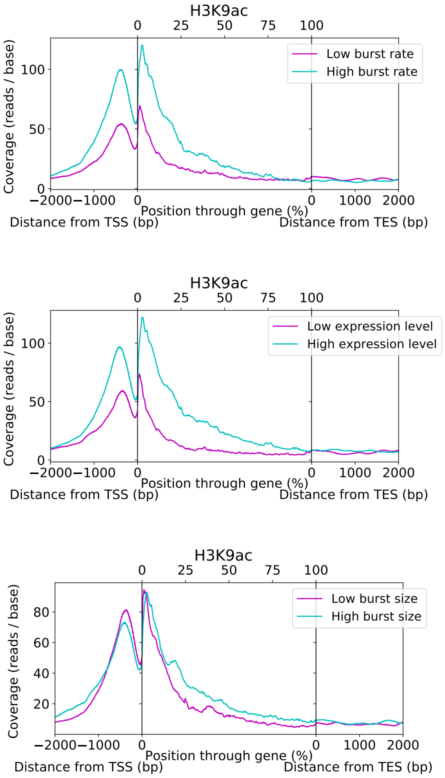

Figure S7: Metagene plots of H3K9ac coverage, comparing profiles for the top and bottom 50% of genes when split according to their estimates for each parameter, denoted by high and low, as indicated.

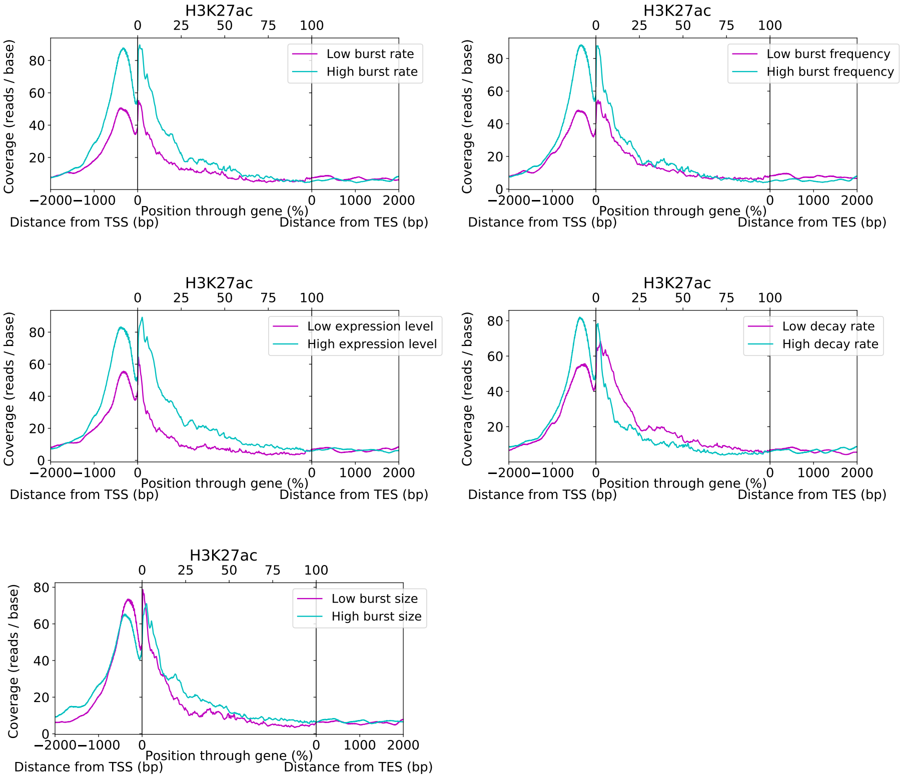

Figure S8: Metagene plots of H3K27ac coverage, comparing profiles for the top and bottom 50% of genes when split according to their estimates for each parameter, denoted by high and low, as indicated.

Gene body-localised histone modifications

A similar analysis of the GB-localised HMs was also carried out, where we use H3K36me3 as a representative example, although their metagene profiles and bursting associations are somewhat more diverse than with the four promoter-localised HMs. H3K4me1 was categeorised as being primarily promoter associated in [5] but we find its connections to transcriptional dynamics instead to be contingent upon its presence throughout the GB, and have therefore reclassified it for this context. The profiles of H3K36me3 halved by the different bursting

parameter estimates as before (figure S9) indicate that presence throughout the GB and around the TES seems to be associated with increased μ, a and κ in a uniform manner. No association with b or δ is apparent, suggesting that this HM is associated with increased μ purely through increased κ. In this case, we are able to support the previously reported correlation with a [5], and confirm that the inability of scRNA-seq data to distinguish a and κ did not skew the final conclusions by quantifying the strength (figure S10a) and statistical significance (figure S10b) of H3K36me3 and the other GB-localised HMs which it represents (H3K79me2 and H4K20me1). It should be noted, however, that based on the metagene analysis, while both H3K79me2 (figure S11) and H4K20me1 (figure S12) appear to be primarily associated with increased μ, a and κ, along with H3K4me1 (figure S13), they look to have a positive and negative association with b and δ, respectively, when found throughout the 20%:100% region. However, this is statistically significant only for H3K4me1 (figure S10b), which also has no association with κ and no significant correlation with a. Therefore, for the GB-localised HMs, H3K36me3, H3K79me2 and H4K20me1 can be regarded as similar in their associations with bursting dynamics, while H3K4me1 is an outlier. H3K4me1 is known to be strongly associated with enhancers [6] and, therefore, its presence throughout the portion of the GB downstream of the TSS may signify intronic enhancers, which could enable larger bursts for the gene they are contained within. The regions of association for the GB-localised HMs vary to a degree, as dictated by the metagene analysis, with the values used for the correlation analysis shown in figures S10a and S10b being averaged across 0%:2000 (TSS to 2000bp downstream of the TES) for H3K36me3 and H4K20me1, -2000:100% for H3K79me2 and 20%:100% for H3K4me1.

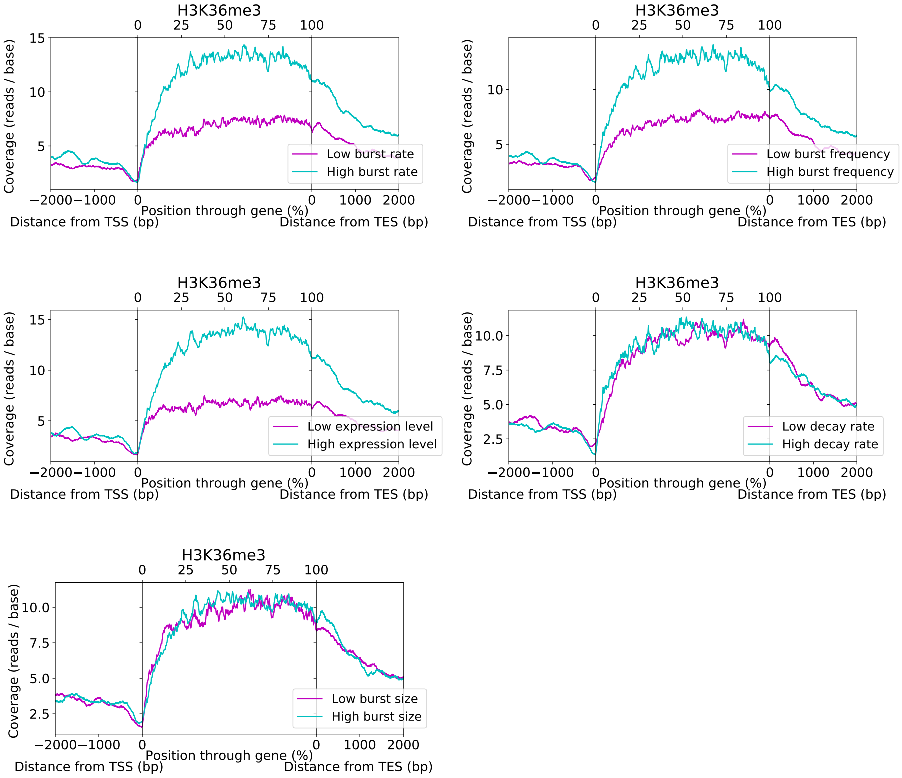

Figure S9: Metagene plots of H3K36me3 coverage, comparing profiles for the top and bottom 50% of genes when split according to their estimates for each parameter, denoted by high and low, as indicated.

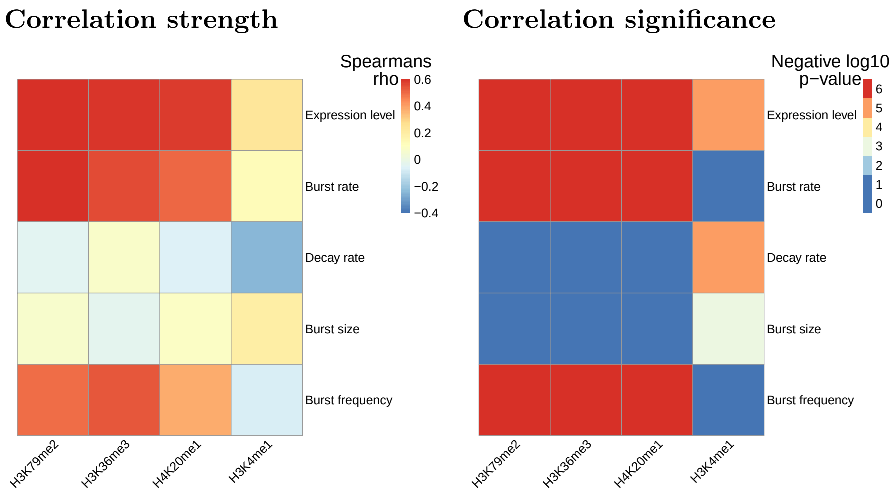

(a) (b)

Figure S10: **a** Heatmap showing the Spearman's rank rho as the heat intensity value for the correlations between bursting parameter estimates and the mean GB-localised HM coverage values across 0%:2000 for H3K36me3 and H4K20me1, -2000:100% for H3K79me2 and 20%:100% for H3K4me1. More intense red or blue colouration indicates a stronger positive or negative correlation, respectively, while neutral indicates no/weak correlation. **b** Heatmap showing the Spearman's rank p-value (adjusted for multiple hypothesis testing) as the heat intensity value for the correlations between bursting parameter estimates and the mean GB-localised HM coverage values across 0%:2000 for H3K36me3 and H4K20me1, -2000:100% for H3K79me2 and 20%:100% for H3K4me1. The heat values are discretised, corresponding to negative log10 p-value thresholds. For example, the most intense blue indicates that, for the given correlation, $10^{-2} < p$, meaning no statistical significance, the neutral colour indicates that $10^{-4} < p \leq 10^{-3}$, while the most intense red indicates that $p \leq 10^{-6}$.

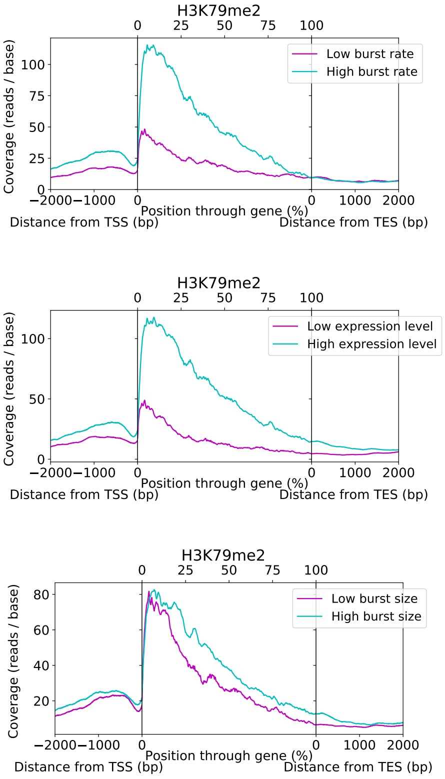

Figure S11: Metagene plots of H3K79me2 coverage, comparing profiles for the top and bottom 50% of genes when split according to their estimates for each parameter, denoted by high and low, as indicated.

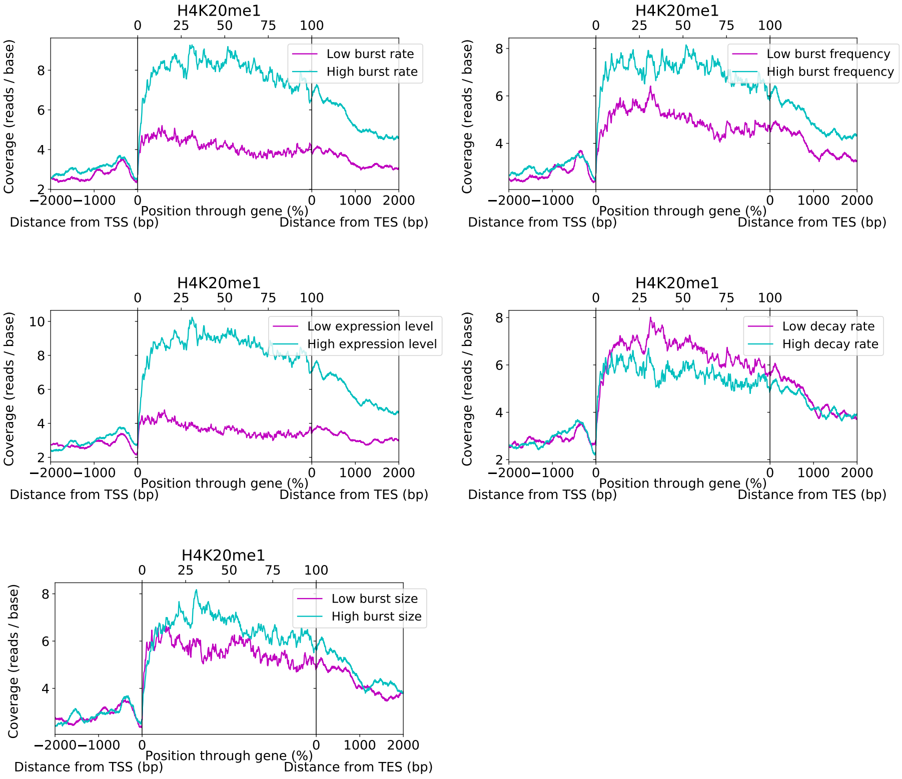

Figure S12: Metagene plots of H4K20me1 coverage, comparing profiles for the top and bottom 50% of genes when split according to their estimates for each parameter, denoted by high and low, as indicated.

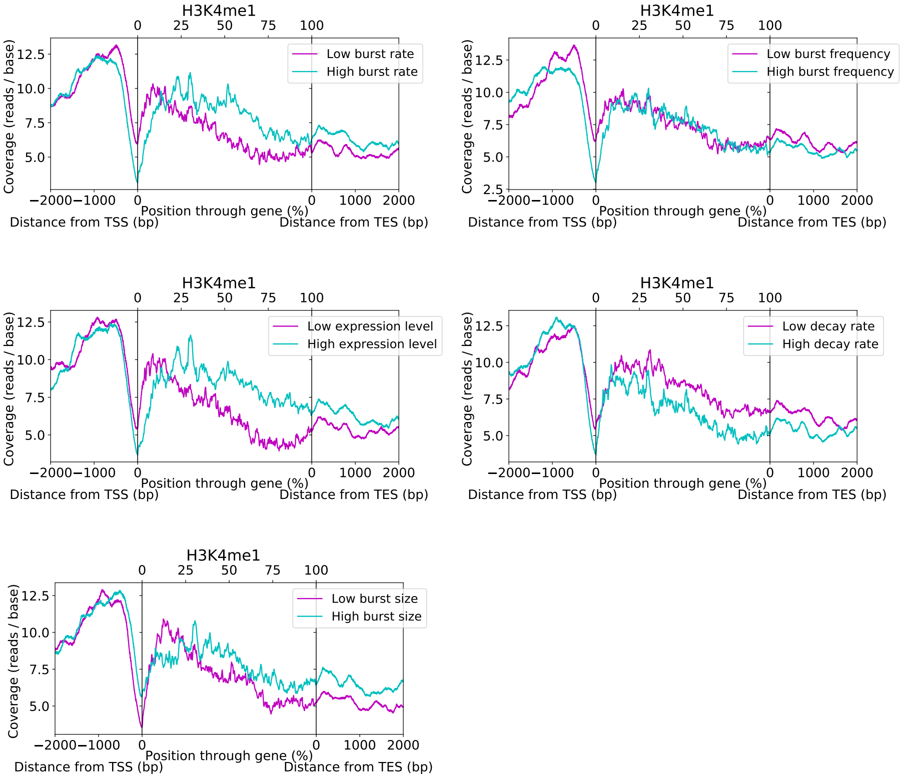

Figure S13: Metagene plots of H3K4me1 coverage, comparing profiles for the top and bottom 50% of genes when split according to their estimates for each parameter, denoted by high and low, as indicated.

Additional histone modifications

Analysis of two additional HMs was carried out based on previous work strongly linking them to active enhancer regions [6], which were not analysed in [5]. The metagene for H4K16ac (figure S14) indicates an association with increased burst rate by increased burst frequency, which overcomes an increased decay rate. Likewise, the increased burst rate overcomes the reduced burst size to result in overall increased expression level. The effects which would reduce gene activity (b and δ) begin at the TSS and appear to grow in strength throughout the

GB and towards the TES, eventually neutralising the κ-related positive effect on expression which is uniform throughout the GB and begins upstream of the TSS. On the other hand, the metagene for H3K18ac (figure S15) indicates a positive association with expression level and burst size, along with a possible negative association with decay rate, but no effect related to burst frequency. We next quantify the strength of the correlations between the HM coverage values and parameter estimates (figure S16a) and their statistical significance (figure S16b), confirming the significance of the positive association of H3K18ac with both expression level and burst size over the -2000:100% region, but not with decay rate. For H4K16ac, we find significant positive associations with expression level, burst rate and burst frequency over the -2000:100% region, and significant positive and negative associations with decay rate and burst size, respectively, over the 0%:+2000 region.

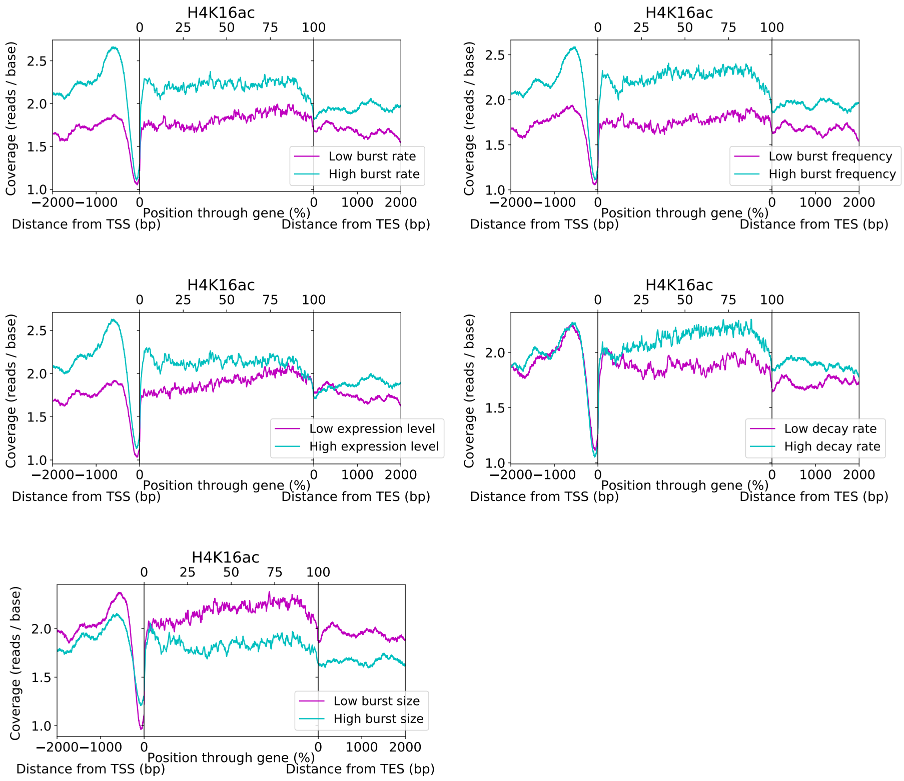

Figure S14: Metagene plots of H4K16ac coverage, comparing profiles for the top and bottom 50% of genes when split according to their estimates for each parameter, denoted by high and low, as indicated.

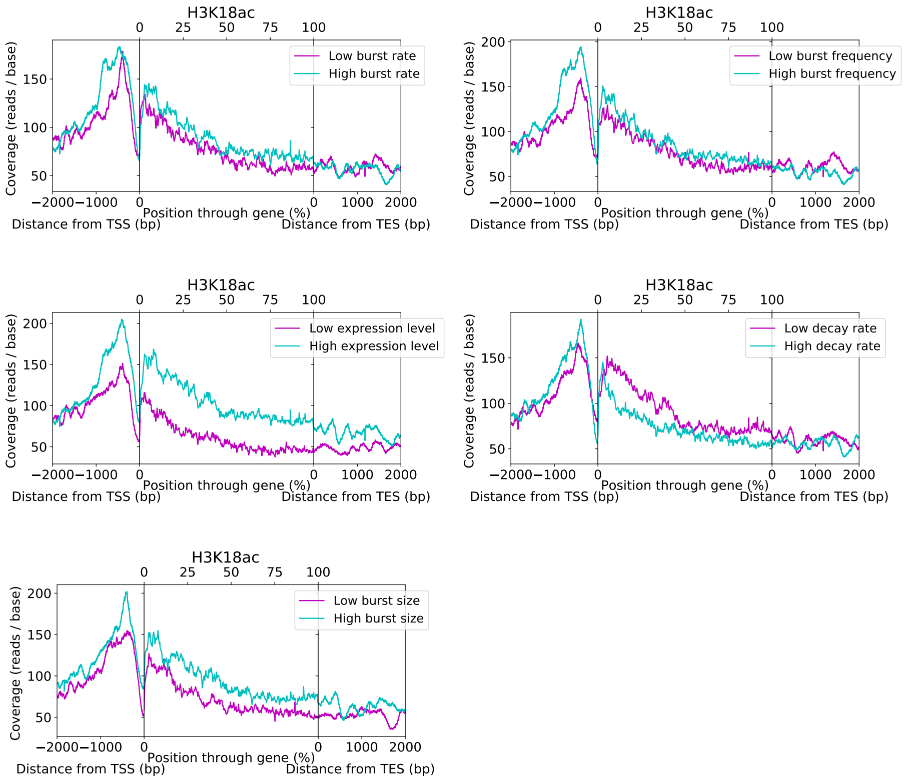

Figure S15: Metagene plots of H3K18ac coverage, comparing profiles for the top and bottom 50% of genes when split according to their estimates for each parameter, denoted by high and low, as indicated.

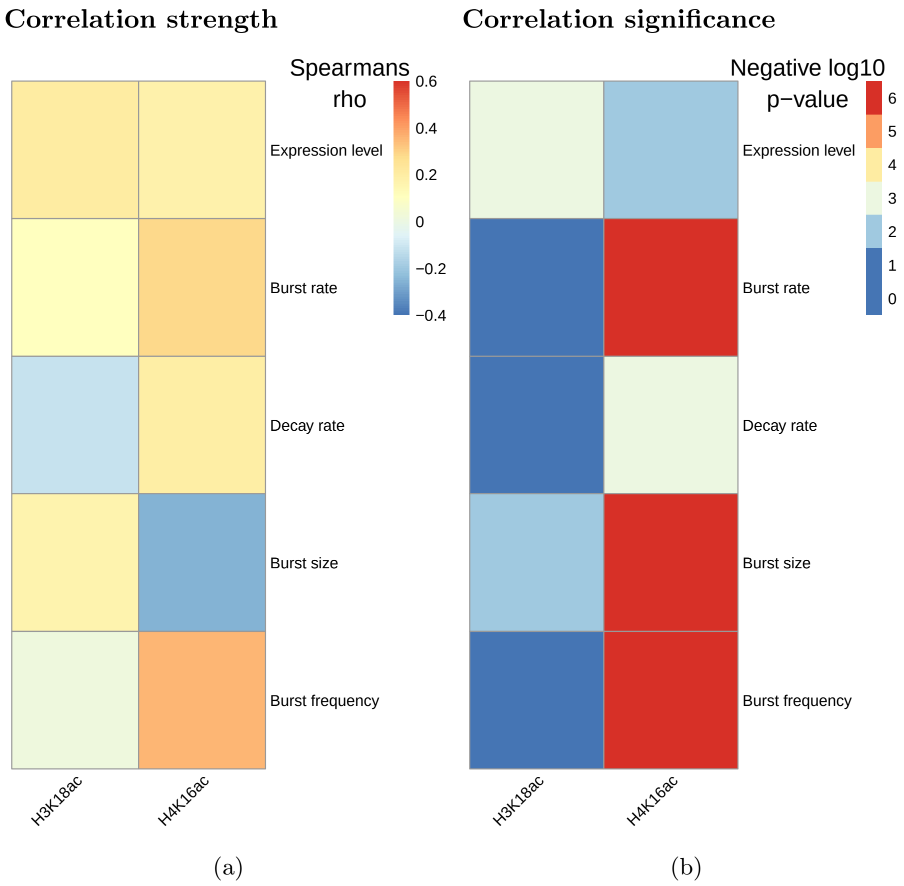

Figure S16: **a** Heatmap showing the Spearman's rank rho as the heat intensity value for the correlations between bursting parameter estimates and the mean HM coverage values across -2000:100% (μ, a and κ) or 0%:2000 (b and δ) for H4K16ac and across -2000:100% for H3K18ac (all parameters). More intense red or blue colouration indicates a stronger positive or negative correlation, respectively, while neutral indicates no/weak correlation. **b** Heatmap showing the Spearman's rank p-value (adjusted for multiple hypothesis testing) as the heat intensity value for the correlations between bursting parameter estimates and the mean HM coverage values across -2000:100% (μ, a and κ) or 0%:2000 (b and δ) for H4K16ac and across -2000:100% for H3K18ac (all parameters). The heat values are discretised, corresponding to negative log10 p-value thresholds. For example, the most intense blue indicates that, for the given correlation, $10^{-2} < p$, meaning no statistical significance, the neutral colour indicates that $10^{-4} < p \leq 10^{-3}$, while the most intense red indicates that $p \leq 10^{-6}$.

References

[1] Klein AM, Mazutis L, Akartuna I, Tallapragada N, Veres A, Li V, et al. Droplet barcoding for single-cell transcriptomics applied to embryonic stem cells. Cell. 2015;161(5):1187–1201.

[2] Wang J, Huang M, Torre E, Dueck H, Shaffer S, Murray J, et al. Gene expression distribution deconvolution in single-cell RNA sequencing. Proceedings of the National Academy of Sciences. 2018;115(28):E6437–E6446.

[3] Schofield JA, Duffy EE, Kiefer L, Sullivan MC, Simon MD. TimeLapse-seq: adding a temporal dimension to RNA sequencing through nucleoside recoding. Nature methods. 2018;15(3):221.

[4] Roberts GO, Rosenthal JS. Optimal scaling of discrete approximations to Langevin diffusions. Journal of the Royal Statistical Society: Series B (Statistical Methodology). 1998;60(1):255–268.

[5] Wu S, Li K, Li Y, Zhao T, Li T, Yang YF, et al. Independent regulation of gene expression level and noise by histone modifications. PLoS computational biology. 2017;13(6):e1005585.

[6] Wolfe JC, Mikheeva LA, Hagras H, Zabet NR. An explainable artificial intelligence approach for decoding the enhancer histone modifications code and identification of novel enhancers in Drosophila. Genome Biology. 2021;22(1):1–23.
