# Supplementary figures and images for: Synergising single-cell resolution and 4sU labelling boosts inference of transcriptional bursting
Source: Genome Biol. 2023 Jun 16;24:138. doi: 10.1186/s13059-023-02977-y (PMC10276402; doi:10.1186/s13059-023-02977-y)

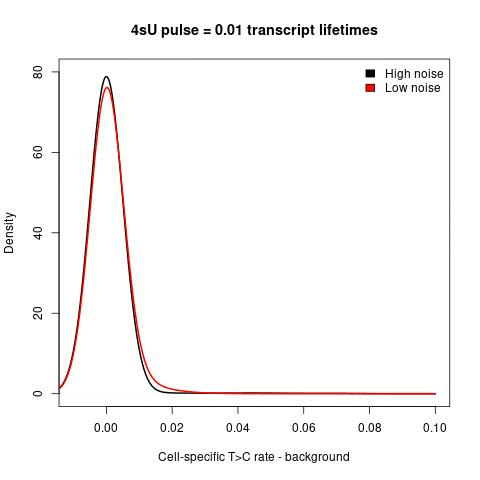

Supplement: Supplementary file 2 — Additional file 2. High vs low noise cell-specific T>C rate distribution transition: Video gif showing the differential transition from surviving to new transcript pool for high and low noise genes through the cell-specific T>C rate distributions for data simulated with different pulse durations. [file 13059_2023_2977_MOESM2_ESM.gif]
