# Supplementary material for: Synergising single-cell resolution and 4sU labelling boosts inference of transcriptional bursting
Source: Genome Biol. 2023 Jun 16;24:138. doi: 10.1186/s13059-023-02977-y (PMC10276402; doi:10.1186/s13059-023-02977-y)
Supplement: Supplementary file 3 — Additional file 3. Review history. [file 13059_2023_2977_MOESM3_ESM.docx]

Review History

**First round of review**

**Reviewer 1**

**Were you able to assess all statistics in the manuscript, including the appropriateness of statistical tests used?**

Yes: all methods appropriate

**Were you able to directly test the methods?**

No

**Comments to author:**

Edwards et al., present a new method to analyse single cell transcriptomics data with 4sU labelling. This is a new technology and novel methods to analyse this type of data are indeed needed. In particular, the authors show how their method can infer transcription dynamics parameters from 4sU labelled scRNA-seq datasets. Interestingly, they found that high expression levels are achieved through increased burst size, not increased burst frequency or reduced decay rate. They also identified a very important correlation between burst frequency and decay rate. Finally, they used histone modification datasets to investigate the relationship between epigenetics and burst parameters. Most importantly, promoter-localised histone modifications are only associated with increased burst frequency when found around the TSS, while their presence further downstream remains associated with increased burst rate, and therefore reduced transcriptional noise. Overall, I found the method useful and the results interesting and, thus, I recommend publication of this paper in Genome Biology, subject to a few straightforward changes.

The main issue is that the code to run the analysis is not available. I recommend the authors to make the scripts available at least with comments and examples on github. Ideally, they should make an R package, which I think will significantly increase the use of their method and future citations.

There are several minor points that I think the authors should address.
1. For the chemical reaction scheme, the authors should also cite older work https://royalsocietypublishing.org/doi/abs/10.1098/rsif.2009.0474 which also showed that mRNA degradation has a high contribution to noise in gene expression.
2. We know TFs performed facilitated diffusion that can impact with modelling activation. Nevertheless, one can model activation as a 2-state process and this was shown previously in https://journals.aps.org/pre/abstract/10.1103/PhysRevE.90.032701). Authors should mention this.
3. I would recommend the authors to also look at the relationship of H3K18ac, H4K16ac and H3K79me3 and bursting since these marks have been previously shown to be involved in active regulatory regions (https://genomebiology.biomedcentral.com/articles/10.1186/s13059-021-02532-7)
4. Having methods after results for this paper makes the reading challenging. Nevertheless, I understand if this is a requirement of the journal.

**Reviewer 2**

**Were you able to assess all statistics in the manuscript, including the appropriateness of statistical tests used?**

Yes: statistics used is appropriate.

**Were you able to directly test the methods?**

No

**Comments to author:**

The paper under review presents a novel method for performing inference of transcriptional burst kinetics from single-cell 4sU-labelled RNA-sequencing data. The manuscript argues that, despite several single-cell RNA labelling approaches having been developed to resolve transcriptome dynamics, such as synthesis, splicing and degradation rates of mRNA, these methods have not been fully exploited for understanding transcriptional bursting. A new methodology is presented that shows how combining high-throughput resolution of scRNA-seq with 4sU-labelling can characterise transcriptional burst dynamics at genome-wide scale. The authors present a mathematical model and an adaptive MCMC inference framework to infer the bursty kinetic parameters from 4sU scRNA-seq data. They perform model comparison by testing the recovery of known parameters from synthetic data by three different likelihood functions: the L1 likelihood models the conventional scRNA-seq UMI counts, the L2 likelihood models the single-cell T>C conversions of the labelling protocol, and a third L1 + L2 likelihood incorporates both to model 4sU scRNA-seq data. The performance of these three models on simulated data indicates that the third model can be used to infer bursting kinetics from real data. In this manuscript, transcriptional bursting at the single gene level is understood through a set of kinetic rates: burst size b, burst frequency κ, decay rate δ, burst rate a = κ/δ and expression level μ = b × a. The authors show that, even though scRNA-seq can be used to infer a, b and μ, the estimates are more robust if the 4sU conversion data are included in the inference. At the same time, scRNA-seq data alone are not sufficient to estimate κ and δ, but including conversion data can result in accurate estimation of these parameters. The authors apply inference to all genes from the dataset published by Qiu et al. (2020), that have at least one read and T>C conversion, and they select a final set of 606 genes, for which they achieve robust estimation of parameters (coefficient of variation of estimates is below a certain threshold). A hybrid inference approach is applied for a certain set of genes that lie in extreme regions of the state space, which combines the analytical likelihood with simulation-based inference. Downstream analysis of the selected genes reveals patterns of how parameter estimates across genes depend on their CV (the degree of confidence), as well as how the kinetic rates correlate with statistics of the data. Additionally, the performance of the inference framework is tested against synthetic data, showing strong correlations between ground-truth and estimated parameters. The analysis is also focused on a pair of genes (ATF5 and TSN) with similar mean expression and decay rates but different burst kinetics, ATF5 being more bursty. The authors explain how the difference in the genes' distributions of T>C rates, demonstrates that 4sU labelling can identify between different transcriptional bursting patterns and their cell-to-cell variation. This argument is reinforced by simulated data of genes with different burst kinetics, where the effect of cell-specific capture efficiency of the real data is removed. The authors provide some biological conclusions that arise from their inference results. They support that high gene expression levels can be primarily explained by large burst size rather than large burst frequency, while the decay rates appear to be well constrained. They also argue that positive correlations between κ and δ might indicate that cells control the levels of transcriptional noise by stabilising burst rates a. Finally, the study explores relationships between histone modifications and transcriptional bursting using ChIP- seq data.

This is an important and timely topic and the paper overall is of interest to biologists and computational biologists. However, the mathematical methods used in the paper can be more clearly presented. I have the following comments on the paper:

Comments

* Methods: Mathematical modelling of transcriptional bursting is based on the standard two-stage tele- graph model. The likelihood of observing l UMIs in a single cell is obtained by compounding the analytical steady-state solution of the telegraph model (negative binomial) with a Poisson distribution (which approximates the probability of observing l UMIs given m total transcripts), given the cell's capture efficiency. What is not clear about this point is why the authors choose to not compound directly with the binomial distribution that models the probability of the true capturing process (see e.g BayNorm Bioinformatics 2020). If the likelihood is analytically intractable when compounding with the binomial, then this could be stated by the authors. The procedure for computing the joint probability distribution of l UMIs and y T>C conversions per read for each single cell is mostly clear. One comment to be made is related to the parameter λn, the gene-invariant conversion rate due to 4sU labelling (see Comments on Appendix).

* A general comment on the overall presentation of the mathematical model and the inference framework: Even though the individual aspects and steps of modelling are described in detail, it is hard for the reader to fully understand the proposed model by reading this section of the methods. This is because some important modelling choices and assumptions are clarified in other parts of the manuscript. Perhaps a summary text - figure that gives an overview of the model would help link all the separate parts together and help the reader understand the general idea (see also Comments on figures).

* A comment on the analytical/numerical hybrid likelihood: In the part of this section where the ABC approach is described, in page 25 of 47, lines 33-34, it is mentioned that the single-cell aspect of the data is neglected and the total number of reads across all cells is drawn from a Poisson distribution. Eventually, the distance between the generated and the real data is based on comparing the cumulative distributions of the total number of reads with i conversions across all cells. This means that the out- come of the ABC acceptance-rejection algorithm depends on a single summary statistic which in a sense neglects/underestimates the effect of cell-to-cell variability. Perhaps, the choice of the authors to choose this specific distance function (and this summary statistic) in their hybrid likelihood should be justified more extensively.

* Appendix: The method for estimating the conversion rates λs and λn is mostly clear. One point that is a bit ambiguous is that for estimating the rate λn from data, the authors initially assume that all reads correspond to new transcripts, synthesised during the labelling pulse. Perhaps the authors could justify this a bit further, as it is quite a strong assumption. Also, λn is estimated uniformly, it is the same across all cells. How possible is it for the value of this parameter to vary due to technical/experimental errors in the protocol (e.g. not uniform labelling of all the cells)? Could this parameter be inferred simultaneously with the kinetic rates?

* The paragraph "Inference on simulated data" in the appendix is overall well written. One unclear point here is why the authors choose to not neglect the single-cell aspect in the simulation of T>C data. How is this choice justified, compared to the choice in the hybrid/simulation-based inference on real data?

* Figures: The pdf given (either due to the submission issues or authors choice, had figures at the end without figure numbers and one had to use file names to match the figures to captions. This should be avoided in the revision, otherwise, I will refuse to review the paper again (as it is extremely time consuming to understand the paper). Also, the paper has 25 figures (10 referenced in results and the rest on method and appendix). I suggest combining figures into fewer main and the rest cold be SI figures. In addition, it would be recommended that the authors create a figure-schematic that illustrates what is the structure of the dataset as well as summarises the main structure of the modelling and inference framework. Even though the methods are explained in detail in different parts of the manuscript, a main figure that summarises and links all information would help the overall understanding of the pipeline. For example, they could include a cartoon illustration of the labelling protocol used to generate the data. On top of that, the figure could illustration how is the information in the data precisely connected with the underlying transcriptional bursting model. What aspects of the 4sU sRNA-seq data are informative for estimating the various kinetic rates of the transcription model?

* Observed typos: At the end of the page 20 of 47, in the last sentence above equation (13), it should say "compounding equations 10 and 12" instead of "compounding equations 9 and 11". Similarly, in page 21 of 47, above equation (14), it should say "determined by equation 13" instead of "determined by equation 12" and above equation (16), it should say "the product of equations 5 and 15" instead of "the product of equations 4 and 14". It is recommended that the whole section of Mathematical modelling and parameter inference in the Methods should be checked for similar typos where the numbering of the equations the text refers to is shifted by 1.

* One comment on the paragraph "Biological findings" in the results, in the second-to-last sentence, the phrase "otherwise too small a proportion may express the gene for the function to be fulfilled" is not clear and could be rephrased.

**Authors Response**

**Point-by-point responses to the reviewers’ comments:**

We greatly appreciate the constructive comments of the reviewers and are thankful for their work. We have now revised and improved our manuscript based on the reviewers’ input, as explained below.

While working on our revision to respond to a reviewer comment (see C4, reviewer 2 below), we noticed a significant improvement that was possible with our approach and implemented this. In a nutshell, we have replaced the “hybrid” inference method, in which we combine an ABC-based evaluation of cell-summed T>C count data and the analytical likelihood 1 assessment of UMI count data, with a fully analytical assessment using “model 3”.

Model 3 combines likelihood 1 with likelihood 3, a new likelihood function analytically modelling cell-summed T>C count data which depends only on δ and not a or b, unlike the likelihood 2 we use in model 2. Model 3 is faster and robust against regions of parameter space which model 2 cannot deal with. Therefore, we no longer need to rely on the hybrid numerical-analytical approach, making the method cleaner, faster, more robust and no longer dependent upon choice of summary statistic or epsilon (distance) value.

Relying exclusively on likelihood functions during inference also allowed implementation of a Metropolis-adjusted Langevin algorithm (MALA) instead of our previous random walk metropolis (RWM) MCMC method. This approach is essentially the same but utilises the likelihood gradient for more efficient and reliable convergence to the target density than is achieved with RWM. More details about it are given in the methods section.

We re-ran our analyses in the results section (and SI) using the improved methods, which produced the same outcomes, apart from minor differences.

Please see below for specific responses to the reviewer comments.

**Reviewer 1**

**C1:** The main issue is that the code to run the analysis is not available. I recommend the authors to make the scripts available at least with comments and examples on github. Ideally, they should make an R package, which I think will significantly increase the use of their method and future citations.

**R1:** The scripts used for running the inference algorithm, including a script which generates a file in the final format to be input into the algorithm from the processed Qiu data, as well as a script for simulating data based on the Qiu data to benchmark algorithm performance, and a readme, have been uploaded to our GitHub page (linked in the paper; https://github.com/hebenstreitLab/burstMCMC).

This has been made into an R package called ‘burstMCMC’ with accompanying sample data for ease of use.

The scripts used for the pre-processing pipeline for the Qiu data and extracting the required information, along with a readme, have also been made public by depositing on our GitHub page (https://github.com/hebenstreitLab/burstMCMCpreprocessing).

**C2:** For the chemical reaction scheme, the authors should also cite older work https://royalsocietypublishing.org/doi/abs/10.1098/rsif.2009.0474 which also showed that mRNA degradation has a high contribution to noise in gene expression.

**R2:** A sentence has been added to the end of the 2^nd^ last paragraph of the introduction (page 5) mentioning the expectation of significant noise contribution by degradation based on this paper, and how it is therefore important to account for when looking at bursting. Another sentence has been added to the 2^nd^ paragraph of the discussion (page 20) linking these predictions with our findings.

**C3:** We know TFs performed facilitated diffusion that can impact with modelling activation. Nevertheless, one can model activation as a 2-state process and this was shown previously in https://journals.aps.org/pre/abstract/10.1103/PhysRevE.90.032701). Authors should mention this.

**R3:** While introducing the two-state model at the start of the “Mathematical modelling” section of the methods, we have included additional text about facilitated diffusion of TFs and how the two-state model still works due to high DNA sliding speeds observed, as shown in this cited paper.

**C4:** I would recommend the authors to also look at the relationship of H3K18ac, H4K16ac and H3K79me3 and bursting since these marks have been previously shown to be involved in active regulatory regions (https://genomebiology.biomedcentral.com/articles/10.1186/s13059-021-02532-7).

**R4:** Thank you for this suggestion. We downloaded datasets for H3K18ac and H4K16ac (no cell-matched data for H3K79me3) from <https://www.ncbi.nlm.nih.gov/geo/query/acc.cgi?acc=GSM2858421> <https://www.ncbi.nlm.nih.gov/geo/query/acc.cgi?acc=GSM2862934> <https://www.ncbi.nlm.nih.gov/geo/query/acc.cgi?acc=GSM4809274>
and did the metagene/correlation analysis for those too, only using the better quality H3K18ac replicate dataset. The analysis seems to have yielded quite interesting results which can be found in the SI (referenced in results) in three additional figures at the end, along with citation of this paper which the analysis was based on.

**C5:** Having methods after results for this paper makes the reading challenging. Nevertheless, I understand if this is a requirement of the journal.

**R5:** Unfortunately, this is indeed a requirement of the journal, otherwise we would have been happy to move the methods before the results.

**Reviewer 2**

**C1:** Mathematical modelling of transcriptional bursting is based on the standard two-stage telegraph model. The likelihood of observing l UMIs in a single cell is obtained by compounding the analytical steady-state solution of the telegraph model (negative binomial) with a Poisson distribution (which approximates the probability of observing l UMIs given m total transcripts), given the cell's capture efficiency. What is not clear about this point is why the authors choose to not compound directly with the binomial distribution that models the probability of the true capturing process (see e.g BayNorm Bioinformatics 2020). If the likelihood is analytically intractable when compounding with the binomial, then this could be stated by the authors.

**R1:** The Poisson distribution is used for capture efficiency modelling instead of the true binomial distribution because it permits the upper limit M value to be lower than l (UMI count) without making the likelihood zero, allowing for a likelihood gradient rather than a flat zero across regions of parameter space with too low expression level or M value.

This lets the Markov chain escape such regions much more efficiently by following the gradient and converge to the target density quickly without having a high chance of getting stuck. The approximation is valid for low alpha such as in the Qiu dataset.

A sentence briefly explaining this has been added below equation 3:
“The approximation in equation 2 allows non-zero probability values even when m < l, which allows our MCMC algorithm to more efficiently escape regions of parameter space for which M < l.”

**C2:** The method for estimating the conversion rates λs and λn is mostly clear. One point that is a bit ambiguous is that for estimating the rate λn from data, the authors initially assume that all reads correspond to new transcripts, synthesised during the labelling pulse. Perhaps the authors could justify this a bit further, as it is quite a strong assumption. Also, λn is estimated uniformly, it is the same across all cells. How possible is it for the value of this parameter to vary due to technical/experimental errors in the protocol (e.g. not uniform labelling of all the cells)? Could this parameter be inferred simultaneously with the kinetic rates?

**R2:** By assuming that all reads come from new transcripts we really assume that at least a small subset of genes have a high enough δ to fully (or very nearly) turn over their transcriptomes across all cells. Only the genes with the very highest background-subtracted T>C rate will actually impact the inferred λn value which corresponds to the maximum achievable 4sU-mediated T>C rate i.e., when all reads are new. Genes with lower proportions of new transcripts have no effect on inferring this value since we use the survival function of the binomial distribution,
P(X > TC – 1 | T, λn + λs), meaning that the lower the T>C rate of a gene the higher the probability of observing that many TCs or more. In this way we only really assume a small subset of genes have 100% new transcripts. An explanation of this has been added to the end of the “Conversion rates” section in the SI.

A small subset (>=10) exhibiting complete turnover is highly likely considering the 4 hour 4sU pulse used in the Qiu data, and is strongly supported by the shape of figure 11 (previously figure 14), which shows the control vs 4sU T>C rates. The shape of the cloud of points is truncated at the top where the upper bound is, being flat with a high density of points there. If no genes had complete turnover then we would expect the cloud of points to thin out towards the top in a manner more similar to the bottom of the cloud, rather than the sharp truncation which is observed. This justification has been included in the aforementioned explanation in the manuscript.

Regarding the suggestion to infer λn in a cell-specific manner, this could be something good to aim for in the future as there does seem to be a degree of variation between cells (although this can also be influenced by which sets of genes are being expressed), however there are a few things which make this impractical:

Firstly, by relying on the data associated with single cells rather than the collective dataset, we reduce the number of genes with high enough confidence in the T>C rate which we can use to infer λn from 6307 when including all cells to 158 on average when calculating a separate λn for each cell individually, with some cells even having <10 such genes due to having less data overall and about half having <100. This would make it impossible to robustly estimate λn for a significant number of cells because we cannot guarantee that any high confidence genes have undergone complete turnover.

Secondly, the algorithm uses matrix operations to efficiently calculate distributions, including the probability distribution of i conversions (rows) in a given read conditional on m transcripts in the cell (columns), which is not immediately compatible with cell-specific λn. Therefore, this part of the algorithm would need to be adjusted and would likely be slower.

Finally, inferring λn simultaneously with the biological parameters would be difficult because the algorithm is currently parallelised over each gene, with one Markov chain per gene, only needing the data corresponding to that gene, whereas we would need to run one Markov chain with all biological parameters for all genes and λn simultaneously using the entire dataset since λn is not gene-specific. This would make the algorithm much harder to parallelise and therefore slower, one would lose the convenience of being able to do parameter inference on only a subset of genes of interest, and we would suffer strongly from the “curse of dimensionality” in this scenario, especially with cell-specific λn, in which the dimension is too high for the Markov chain to converge and mix within a reasonable time.

**C3-A:** A general comment on the overall presentation of the mathematical model and the inference framework: Even though the individual aspects and steps of modelling are described in detail, it is hard for the reader to fully understand the proposed model by reading this section of the methods. This is because some important modelling choices and assumptions are clarified in other parts of the manuscript. Perhaps a summary text - figure that gives an overview of the model would help link all the separate parts together and help the reader understand the general idea (see also Comments on figures).

**C3-B:** It would be recommended that the authors create a figure-schematic that illustrates what is the structure of the dataset as well as summarises the main structure of the modelling and inference framework. Even though the methods are explained in detail in different parts of the manuscript, a main figure that summarises and links all information would help the overall understanding of the pipeline. For example, they could include a cartoon illustration of the labelling protocol used to generate the data. On top of that, the figure could illustration how is the information in the data precisely connected with the underlying transcriptional bursting model. What aspects of the 4sU sRNA-seq data are informative for estimating the various kinetic rates of the transcription model?

**R3:** We have now added a schematic (figure 9) showing the observed data and hidden distributions of the model and how they are linked with the various parameters in a cascading fashion, along with which of these are relevant for model 1 vs 2, with an explanatory legend. We have also included a cartoon illustration (figure 8b) of the 4sU incorporation, conversion step and sequencing protocol alongside the two-state bursting model diagram.

**C4:** A comment on the analytical/numerical hybrid likelihood: In the part of this section where the ABC approach is described, in page 25 of 47, lines 33-34, it is mentioned that the single-cell aspect of the data is neglected and the total number of reads across all cells is drawn from a Poisson distribution. Eventually, the distance between the generated and the real data is based on comparing the cumulative distributions of the total number of reads with i conversions across all cells. This means that the outcome of the ABC acceptance-rejection algorithm depends on a single summary statistic which in a sense neglects/underestimates the effect of cell-to-cell variability. Perhaps, the choice of the authors to choose this specific distance function (and this summary statistic) in their hybrid likelihood should be justified more extensively.

**R4:** The choice of summary statistic for quantifying the distance between the data in the hybrid method is no longer an issue since we now have a fully analytical approach using model 3, which is one of the advantages of the updated methodology.

**C5:** The paragraph "Inference on simulated data" in the appendix is overall well written. One unclear point here is why the authors choose to not neglect the single-cell aspect in the simulation of T>C data. How is this choice justified, compared to the choice in the hybrid/simulation-based inference on real data?

**R5:** The only reason we neglected the single-cell aspect of the T>C data in the simulations for our hybrid inference is because it was much easier to derive a summary statistic for the distance between the real and simulated data during Markov chain progression for pinning down δ, and because repeatedly simulating single-cell T>C data can be quite slow depending on the biological parameters, whereas simulating cell-summed T>C data was consistently fast.

When generating simulated data to carry out inference on, the aim is to benchmark the performance of our algorithm on equivalent artificial 4sU scRNA-seq data with known, ground truth parameter values to see if we can recover them. This includes testing performance of inference with model 2 and so requires single-cell T>C count data rather than cell-summed data. Not neglecting the single-cell aspect in the simulation is really the default and we only neglect out of necessity.

Of course, while doing inference on simulated data is still a very important part of our paper, we no longer make use of the hybrid inference approach in favour of model 3, meaning that we no longer do any simulations which neglect the single-cell aspect of the T>C count data.

**C6:** The pdf given (either due to the submission issues or authors choice, had figures at the end without figure numbers and one had to use file names to match the figures to captions. This should be avoided in the revision, otherwise, I will refuse to review the paper again (as it is extremely time consuming to understand the paper). Also, the paper has 25 figures (10 referenced in results and the rest on method and appendix). I suggest combining figures into fewer main and the rest cold be SI figures.

**R6:** Figures and tables are now all embedded within the main text and are properly numbered so that they do not need to be referred to by file names. We have also combined three pairs of previously separate figures to reduce the main text figure count as subfigures of figures 1, 5 and 7. This leaves 7 figures in the introduction/results, with 2 in the methods (including the cartoon illustration and the schematic) and the rest in the SI. A fourth pair in the SI have also been combined as subfigures of figure 19.

**C7:** Observed typos: At the end of the page 20 of 47, in the last sentence above equation (13), it should say "compounding equations 10 and 12" instead of "compounding equations 9 and 11". Similarly, in page 21 of 47, above equation (14), it should say "determined by equation 13" instead of "determined by equation 12" and above equation (16), it should say "the product of equations 5 and 15" instead of "the product of equations 4 and 14". It is recommended that the whole section of Mathematical modelling and parameter inference in the Methods should be checked for similar typos where the numbering of the equations the text refers to is shifted by 1.

**R7:** Thank you for spotting the typos. The in-text referencing of numbered equations has now been automated, rectifying all such instances of incorrect numbering. We say “the product of equations 4 and 15” above equation 16 now because equation one was duplicated in the initial submission (equations 1 and 2 were identical), so now equation 5 in the previous version becomes equation 4 in the new, but 15 is still 15 because an extra equation has been inserted (equation 8), which is an approximate solution allowing for much faster computation of the surviving transcript distribution when the state space is very large.

**C8:** One comment on the paragraph "Biological findings" in the results, in the second-to-last sentence, the phrase "otherwise too small a proportion may express the gene for the function to be fulfilled" is not clear and could be rephrased.

**R8:** We have rephrased the relevant portion of the “Biological findings” results subsection, which has hopefully made it clearer and more straightforward:
“Estimates for κ and δ are also positively correlated, despite κ and δ varying across several orders of magnitude. This correlation may be the result of a selective pressure to limit the variability of a and, for example, prevent transcriptional noise levels from becoming excessively high.”

**Second round of review**

**Reviewer 1**

The authors have addressed all my comments, which improved significantly the manuscript. I recommend publication of this manuscript.

**Reviewer 2**

The authors have addressed my comments, the results and the figures have improved and have been clarified. I like the new illustrations. I am happy for the paper to be accepted.
